# BioNeuralNet: a graph neural network based Multi-Omics network data analysis tool

**DOI:** 10.1093/bioinformatics/btag365

**Published:** 2026-06-10

**Authors:** Vicente Ramos, Sundous Hussein, Mohamed Abdel-Hafiz, Arunangshu Sarkar, Weixuan Liu, Katerina J Kechris, Russell P Bowler, Leslie Lange, Farnoush Banaei-Kashani

**Affiliations:** Department of Computer Science and Engineering, University of Colorado Denver, Denver, CO, United States; Department of Computer Science and Engineering, University of Colorado Denver, Denver, CO, United States; Department of Computer Science and Engineering, University of Colorado Denver, Denver, CO, United States; Department of Biostatistics and Informatics, University of Colorado Anschutz Medical Campus, Aurora, CO, United States; Department of Biostatistics and Informatics, University of Colorado Anschutz Medical Campus, Aurora, CO, United States; Department of Biostatistics and Informatics, University of Colorado Anschutz Medical Campus, Aurora, CO, United States; Genomic Medicine Institute, Cleveland Clinic Main Campus, Cleveland, OH, United States; Division of Biomedical Informatics and Personalized Medicine, University of Colorado Anschutz Medical Campus, Aurora, CO, United States; Department of Computer Science and Engineering, University of Colorado Denver, Denver, CO, United States

## Abstract

**Summary:**

Multi-omics data offer unprecedented insights into complex biological systems, yet their high dimensionality, sparsity, and intricate interactions pose significant analytical challenges. Network-based approaches have advanced multi-omics research by effectively capturing biologically relevant relationships among molecular features (e.g., genes, proteins, metabolites). While these methods are powerful for representing molecular interactions, there remains a need for tools specifically designed to effectively utilize these network representations across diverse downstream analyses. To fulfill this need, we introduce *BioNeuralNet*, a flexible and modular Python framework tailored for end-to-end network-based multi-omics data analysis. *BioNeuralNet* leverages Graph Neural Networks (GNNs) to learn biologically meaningful low-dimensional representations from multi-omics networks, converting these complex molecular networks into versatile embeddings. *BioNeuralNet* supports all major stages of multi-omics network analysis, including several network construction techniques, generation of low-dimensional representations, and a broad range of downstream analytical tasks. Its extensive utilities, including diverse GNN architectures, and compatibility with established Python packages (e.g., scikit-learn, PyTorch, NetworkX), enhance usability and facilitate quick adoption. *BioNeuralNet* is an open-source, user-friendly, and extensively documented framework designed to support flexible and reproducible multi-omics network analysis in precision medicine.

**Availability and implementation:**

The *BioNeuralNet* library is available via The Python Package Index (PyPI). Source code, documentation, tutorials, and workflows are hosted at https://bioneuralnet.readthedocs.io. Code archived at https://doi.org/10.5281/zenodo.17503083.

## 1 Introduction

Recent advancements in multi-omics technologies have facilitated simultaneous profiling of genomics, transcriptomics, proteomics, and metabolomics, significantly deepening our understanding of complex biological systems. Yet, effectively extracting actionable insights from these high-dimensional, sparse datasets remains challenging due to intricate molecular interactions and inherent variability.

Network-based approaches, such as Weighted Gene Co-expression Network Analysis (WGCNA) (Langfelder and Horvath 2008) and Sparse Multiple Canonical Correlation Network (SmCCNet) (Liu *et al.* 2024), have been instrumental in identifying biological modules and key molecular interactions. While these methods effectively capture molecular relationships, they are not always optimized for translating those networks into actionable data representations for downstream tasks. There is a growing need for tools that can build upon these network representations, unlocking their potential for flexible, scalable, and diverse analytical applications.

Building upon the strengths of multi-omics networks, we introduce BioNeuralNet, a flexible, modular Python framework leveraging Graph Neural Networks (GNNs) to transform multi-omics networks into biologically meaningful low-dimensional embeddings. These embeddings distill complex, nonlinear molecular relationships into compact vectorized representations, enabling effective and efficient implementation of a wide range of downstream tasks such as disease prediction, biomarker discovery, and subject-level profiling with improved accuracy and scalability.

BioNeuralNet also supports integration of phenotype and clinical data, further enhancing the biological and clinical relevance of the generated embeddings. Its component-based design ensures adaptability to diverse research scenarios, compatibility with existing Python packages (e.g., scikit-learn, NetworkX, Matplotlib), and ease of use through extensive documentation and illustrative examples.

In the subsequent sections, we present the BioNeuralNet architecture, perform a comparative evaluation of its effectiveness against existing leading multi-omics methods, and demonstrate practical applications enabled by BioNeuralNet-generated representations.

## 2 Related work

Existing approaches to multi-omics analysis can be broadly categorized into two groups: non-network-based statistical methods, and task-specific network-based models.

Traditional statistical methods typically represent multi-omics data as high-dimensional tabular matrices. Methods such as Multi-Omics Factor Analysis (MOFA) (Argelaguet *et al.* 2018) extract shared latent structures across omics modalities in an unsupervised manner. While these approaches are effective at capturing global variation, they generally overlook the relationships and interactions between biomolecular entities, resulting in limited biological interpretability and downstream analytical flexibility.

In contrast, several recent methods employ network-based deep learning to explicitly model multi-omics as biological networks for phenotype prediction. Representative examples include MOGONET (Wang *et al.* 2021) and SUPREME (Kesimoglu and Bozdag 2023), which leverage Graph Convolutional Networks (GCNs) to build supervised classifiers. Other recent tools similarly couple fixed architectures to specific biological structures, such as Gene Ontology hierarchies (Lan *et al.* 2025), random-walk embeddings (Scala *et al.* 2024), or cumulant-based statistics (Bose *et al.* 2021). While these methods demonstrate strong predictive performance for specific tasks such as cancer subtype classification, they are often designed as task-specific pipelines, with network structures coupled to specific ontologies or similarity metrics, and they fail to provide the extensibility and modularity needed for broader exploratory analysis, integration of diverse network types, or adaptation to new tasks.

BioNeuralNet differs fundamentally in that it is not a task-specific model, but rather a general, flexible, user-oriented platform for analysis of multi-omics networks. It exposes network construction, GNN embedding, and downstream analysis as decoupled, interchangeable components rather than a fixed pipeline. BioNeuralNet consolidates these capabilities into a single, user-friendly Python framework with a consistent API to streamline network-based multi-omics data analysis.

## 3 System overview

**Figure 1:**
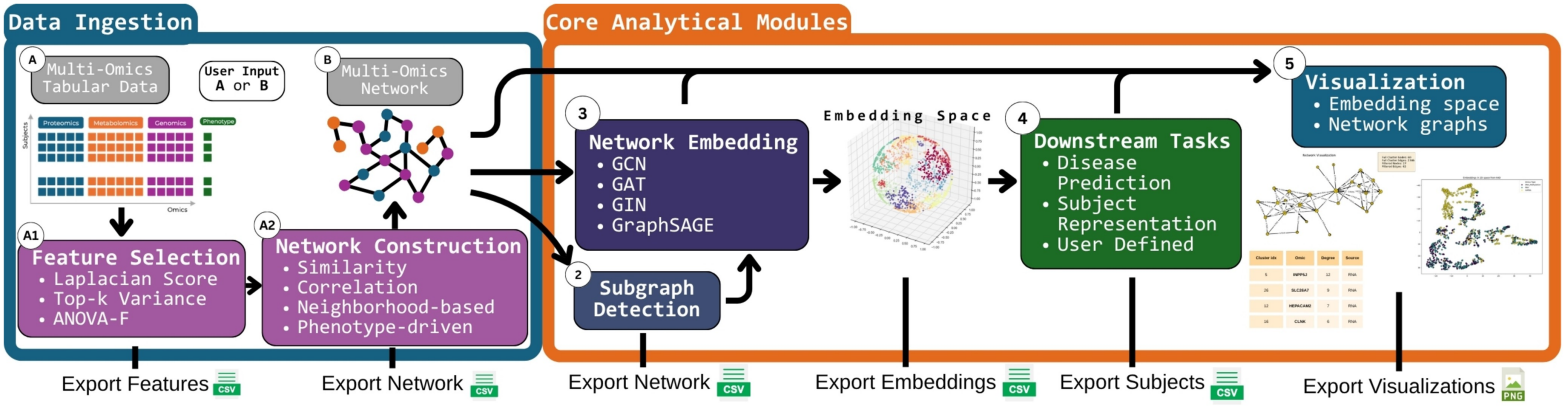
BioNeuralNet workflow overview. Users may begin with either multi-omics tabular data (A) or an existing multi-omics network (B). Tabular data can undergo feature selection (A1) and network construction (A2). The core analytical modules are encapsulated by the orange box. These include optional subgraph detection (2), network embedding using various GNN architectures (3), downstream tasks such as disease prediction (4), and visualizations (5). At each stage of the workflow (A1, A2, 2–5), users can export their outputs, such as constructed networks or GNN embeddings, as CSV files, while visualizations can be saved in PNG format. The embedding space image is adapted from (McInnes *et al*. 2018).

BioNeuralNet is a modular framework that streamlines every stage of network-based multi-omics analysis, from initial data import and network construction to representation learning and downstream analysis (Fig. 1). The system emphasizes flexibility, interoperability, and ease of use, allowing researchers to tailor each step to their experimental context.

### 3.1 Data ingestion

Users can provide their own precomputed multi-omics network, or alternatively upload tabular omics matrices in DataFrame or CSV format, optionally accompanied by phenotype or clinical annotations. For tabular uploads, each omics modality k∈{1,…,K} is represented as a matrix $X^{\left(k\right)}\in R^{n\times p_{k}}$, where *n* is the number of subjects and $p_{k}$ is the number of molecular features. These are concatenated column-wise into a unified multi-omics matrix $X\in R^{n\times p}$, where $p=\sum_{k=1}^{K}p_{k}$ and each element $x_{ij}$ denotes the measurement of feature *j* for subject *i*.

In either case, the resulting network is represented as G=(V,E), where:

-
$V=\{v_{1},\ldots ,v_{p}\}$ is the set of nodes representing the features across all *K* integrated modalities;
-
E⊆V×V is the set of edges describing biological relationships, such as co-expression or similarity;
- Each node $v_{j}$ may carry a feature vector $x_{j}\in R^{n}$ representing molecular measurements across all subjects, while edges in *E* may be weighted to represent the strength or type of association.

Users starting from raw matrices can choose from a variety of network-construction strategies, including *Similarity Networks* (e.g., cosine or Euclidean similarity, with optional pruning and cutoffs), *Correlation Networks* (e.g., Pearson or Spearman correlations, or WGCNA-style soft thresholding), *Neighborhood-based Networks* (e.g., *k*-nearest neighbor), and *Phenotype-driven Networks* (e.g., SmCCNet for supervised multi-omics network inference Liu *et al.* 2024).

The flexibility in data ingestion allows users to benchmark different network topologies and select the approach that best captures the biological relationships within their system of interest. See Availability and Implementation for network construction methods and parameters.

Prior to network construction, BioNeuralNet provides data cleaning and dimensionality reduction for both supervised and unsupervised workflows. Preprocessing utilities include missing value imputation (mean, median, KNN), normalization (standardization, min-max, log_2_), and β-to-M value conversion. Dimensionality reduction is supported via unsupervised filtering (variance thresholding, MAD, PCA, Laplacian Score) and supervised selection (random forest importance, ANOVA-F). See Availability and Implementation for a complete list of preprocessing utilities and examples.

### 3.2 Core analytical modules

#### 3.2.1 Subgraph detection

BioNeuralNet supports both supervised and unsupervised community detection methods to identify biologically meaningful subgraphs within omics networks (Abdel-Hafiz *et al.* 2022). Its core clustering module implements multi-omics extensions of the Louvain algorithm, personalized PageRank, and hybrid approaches, allowing robust detection of dense feature modules associated with phenotypes or experimental conditions. These methods leverage both network connectivity and sample-level data to reveal coherent groups of genes, proteins, or metabolites that may correspond to pathways, functional modules, or disease mechanisms. Output subgraphs can be further analyzed for enrichment, biomarker discovery, or used to reduce dimensionality prior to downstream tasks.

#### 3.2.2 Network embedding

BioNeuralNet supports a range of Graph Neural Network (GNN) architectures for embedding generation, including Graph Convolutional Networks (GCN), Graph Attention Networks (GAT), GraphSAGE, and Graph Isomorphism Networks (GIN) (Scarselli *et al.* 2009). Each model is suited to different types of biological networks and analysis goals. For example, GCNs are effective in the case of uniformly connected graphs, GATs use attention mechanisms to highlight key biological relationships, GraphSAGE is designed for large or dynamic datasets, and GINs are sensitive to subtle feature variations. Users can select from these integrated models within BioNeuralNet or extend the framework with additional architectures that follow the PyTorch Geometric interface (Paszke *et al.* 2019). This modular approach allows embeddings to capture both network structure and biological context, supporting a wide range of downstream analyses.

#### 3.2.3 Downstream tasks

BioNeuralNet enables a broad range of downstream analyses using the generated rich network embeddings, with several key workflows implemented and additional applications easily supported:

- Disease prediction: BioNeuralNet integrates Disease Prediction using Multi-Omics Networks (DPMON) (Hussein *et al.* 2024) for end-to-end supervised disease classification through network embeddings. DPMON combines adjacency networks, multi-omics data, and optional clinical covariates, with support for hyperparameter tuning across GCN, GAT, GraphSAGE, and GIN architectures.
- Enhanced subject representation: BioNeuralNet produces low-dimensional embeddings for each subject leveraging the network-driven representations of the omics instead of the raw omics readings for each sample (Hussein *et al.* 2022). The network-driven subject representation enables enhanced subject subtyping, clustering, biomarker discovery, subject stratification, and visualization.
- Other user-defined tasks: BioNeuralNet embeddings can be used for implementation of a variety of additional downstream tasks, such as omics-omics interaction prediction, functional annotation, pathway discovery, subject similarity analysis, progression trajectory modeling, and comprehensive visualization of network topologies, latent embeddings, and model performance.

By consolidating these components, the framework streamlines the rapid development of new analytical workflows and custom downstream tasks.

## 4 Demonstrative workflow

To illustrate BioNeuralNet’s practical application, we present workflows using two independent multi-omics cohorts (Broad GDAC 2016): (1) TCGA-BRCA breast cancer subtype classification and (2) TCGA-LGG lower grade glioma, a binary survival prediction task (386 alive, 125 deceased). Both cohorts use DNA methylation, mRNA, and miRNA modalities. Analogous analyses for TCGA-KIPAN and other networks are provided in the Supplementary Material. All code and scripts are available in the BioNeuralNet documentation.

### 4.1 Step 1: feature selection

Both cohorts apply unsupervised feature selection independently to each modality matrix $X^{\left(k\right)}$ via the Laplacian Score (He *et al.* 2005), retaining features that best preserve the local manifold structure of the data:


$$L_{r}=\frac{\sum_{ij}{\left(x_{ri}-x_{rj}\right)}^{2}W_{ij}}{\text{Var}(x_{r})}$$

Where $x_{ri}$ and $x_{rj}$ represent the measurements of feature *r* for subjects *i* and *j*, respectively, and $W_{ij}$ is the pairwise similarity between subjects. Lower scores indicate higher importance. We retained 400 DNA methylation, 200 mRNA, and 100 miRNA features per cohort. A full list of available feature selection methods is provided in the Supplementary Material (Page 4, Pipeline Parameters).

### 4.2 Step 2: network construction

Networks were constructed using architecture-paired strategies. For BRCA: a soft-thresholding network paired with GCN and GAT. For LGG: a cosine similarity network paired with GCN, and a Spearman correlation network paired with GAT. Results for additional network-GNN configurations and corresponding ablation studies are detailed in the Supplementary Material (Page 10, Additional Case Studies).

### 4.3 Step 3: GNN-based disease prediction

BioNeuralNet enables end-to-end network-based phenotype prediction by integrating omics data, clinical variables, and network structure within a single, user-friendly function. This is implemented by the DPMON module within BioNeuralNet, which takes as input two components: the subject-level multi-omics data matrix $X_{n\times p}$ and the weighted adjacency matrix $A_{p\times p}$ constructed in Step 2, where *n* denotes the number of subjects and *p* the number of molecular features. DPMON follows a four-step workflow: (1) a GNN extracts informative feature embeddings from multi-omics networks A, capturing both local and global relationships among features, producing a node embedding matrix $E_{p\times d}$, where the embedding dimension *d* is a tunable hyperparameter; (2) dimensionality reduction via a multi-layer autoencoder (reducing the embedding dimension from *d* to 1) is applied to E, yielding a feature importance weight vector w of length *p*, where each entry $w_{j}$ encodes the network-derived importance of feature *j*; (3) the reduced embeddings are integrated with the original multi-omics data through feature weighting, where w scales $X_{n\times p}$ via feature-wise multiplication ($X_{w}=X⊙w^{T}$), producing a weighted matrix $X_{w}$ that enriches each sample’s representation; and (4) a feed-forward neural network classifier takes $X_{w}$ as input to predict the phenotype for each of the *n* subjects, with all components optimized jointly via end-to-end backpropagation. All steps are configurable, allowing users to tailor the analysis while benefiting from BioNeuralNet streamlined workflow.

The results are shown in Tables 1 and 2. On TCGA-BRCA, GNNs perform competitively with classical baselines across all reported metrics. On TCGA-LGG, GCN achieved the highest Recall (0.739±0.050), identifying nearly 10% more mortality cases than the best performing baseline, reflecting the advantage of graph-based modeling on tasks driven by non-linear molecular interactions. Full metrics and additional dataset analyses are provided in the Supplementary Material.

**Table 1:** BioNeuralNet vs. baseline performance on TCGA-BRCA.

Model	Recall	Precision	AUC	AUPR
GCN	0.74±0.07	0.78±0.07	0.95±0.02	0.81±0.05
GAT	0.74±0.05	0.78±0.08	0.95±0.02	0.81±0.04
Log Reg.	0.74±0.05	0.79±0.04	0.96±0.01	0.82±0.04
XGBoost	0.71±0.04	0.81±0.07	0.96±0.01	0.83±0.03
SVM	0.72±0.04	0.76±0.04	0.95±0.02	0.81±0.04

Best results in bold. Full metrics reported to four decimal places (Accuracy, F1-weighted, F1-macro) are available in Table S1.

Results are averaged over 5 runs of 5-fold stratified cross-validation.

**Table 2:** BioNeuralNet vs. baseline performance on TCGA-LGG.

Model	Recall	Precision	AUC	AUPR
GCN	0.74±0.05	0.71±0.03	0.80±0.04	0.55±0.07
GAT	0.70±0.07	0.70±0.05	0.80±0.04	0.58±0.06
Log Reg.	0.64±0.02	0.69±0.02	0.79±0.04	0.55±0.05
XGBoost	0.61±0.05	0.68±0.07	0.76±0.03	0.52±0.04
Rand. For.	0.63±0.04	0.70±0.05	0.73±0.03	0.52±0.06

Best results in bold. Full metrics reported to four decimal places (Accuracy, F1-weighted, F1-macro) are available in Table S2.

Results are averaged over 5 runs of 5-fold stratified cross-validation.

## 5 Conclusion

BioNeuralNet offers a unifying, open-source solution for systems biology, utilizing advanced Graph Neural Networks to tackle the challenges of high-dimensional, sparse datasets. The framework’s robust performance and user-friendly design enable researchers to generate reproducible, scalable, and immediately actionable biological insights necessary for advancements in precision medicine.

## Supplementary Material

btag365_Supplementary_Data

## Data Availability

The preprocessed datasets are incorporated directly into BioNeuralNet and accessed via the bioneuralnet.datasets submodule.
