# Supplementary material for: BioNeuralNet: a graph neural network based Multi-Omics network data analysis tool
Source: Bioinformatics. 2026 Jun 10;42(6):btag365. doi: 10.1093/bioinformatics/btag365 (PMC13293062; doi:10.1093/bioinformatics/btag365)
Supplement: btag365_Supplementary_Data [file btag365_supplementary_data.zip › BioNeuralNet_Supplementary.docx]

**BioNeuralNet Supplementary Materials**

**Table of Contents**

**1. Internal Architecture**

- 1.1 Package Overview
- 1.2 Data Decision Framework & Navigation Guide
- 1.3 Pipeline Parameter Recipes
- 1.4 Automated Hyperparameter Tuning

**2. Methodological Details**

- 2.1 Shared Preprocessing Pipeline
- 2.2 Cohort-Specific Preprocessing Differences
- 2.3 Feature Selection and Laplacian Score
- 2.4 Algorithmic Frameworks

**3. Additional Case Studies**

- 3.1 S1: TCGA-BRCA (Breast Cancer) Subtype Classification
- 3.2 S2: TCGA-LGG (Lower Grade Glioma) Survival Prediction
- 3.3 S3: TCGA-KIPAN (Pan-Kidney) Cancer Staging
- 3.4 S4: ROSMAP (Alzheimer's Disease) Biomarker Discovery

**1.1 Package Overview**

BioNeuralNet is an open-source, modular Python framework tailored for end-to-end network-based multi-omics data analysis. The package architecture is decoupled into functional submodules that mirror the stages of the analytical workflow.

- *datasets*: Manages ingestion via the *DatasetLoader* and provides built-in access to benchmark cohorts including TCGA-BRCA, LGG, KIPAN and synthetic data for experimentation.
- *utils*: Contains several diagnostic and preprocessing tools for data alignment, missing value imputation, and Beta-to-M transformations.
- *feature_selection*: Implements dimensionality reduction strategies such as Laplacian Score, MAD, and Variance Thresholding.
- *network*: Facilitates the construction of multi-omics networks using Similarity, Correlation, and Gaussian KNN strategies.
- *network_embedding*: Generates low-dimensional representations using Graph Neural Network (GNN) architectures including GCN, GAT, SAGE, and GIN.
- *clustering*: Identifies disease-associated subgraphs through hybrid community detection algorithms like Hybrid Louvain and Correlated PageRank.
- *downstream_task*: Executes specialized pipelines including DPMON for phenotype prediction and Subject Representation for patient-level profiling.
- *metrics* & *external_tools*: Provides topological evaluation, visualization utilities, and interoperability with R-based exports.

**1.2 Data Decision Framework & Navigation Guide**

Multi-omics datasets are notoriously high-dimensional, sparse, and noisy. To ensure the pipeline remains robust across different experimental contexts, we provide a structured Data-Driven Decision Flowchart (see image below). This guide serves as a heuristic for navigating the mathematical trade-offs inherent in multi-omics analysis, such as avoiding GNN over-smoothing or network fragmentation.

**Key Decision Heuristics:**

- Preprocessing Quality: Use the data_stats() summary to determine if missingness or bounding issues require specific imputation or transformation actions.
- Feature Scaling: Unsupervised selection (e.g., Laplacian Score) is recommended for high-dimensional layers to isolate biological signal from noise before network construction.
-
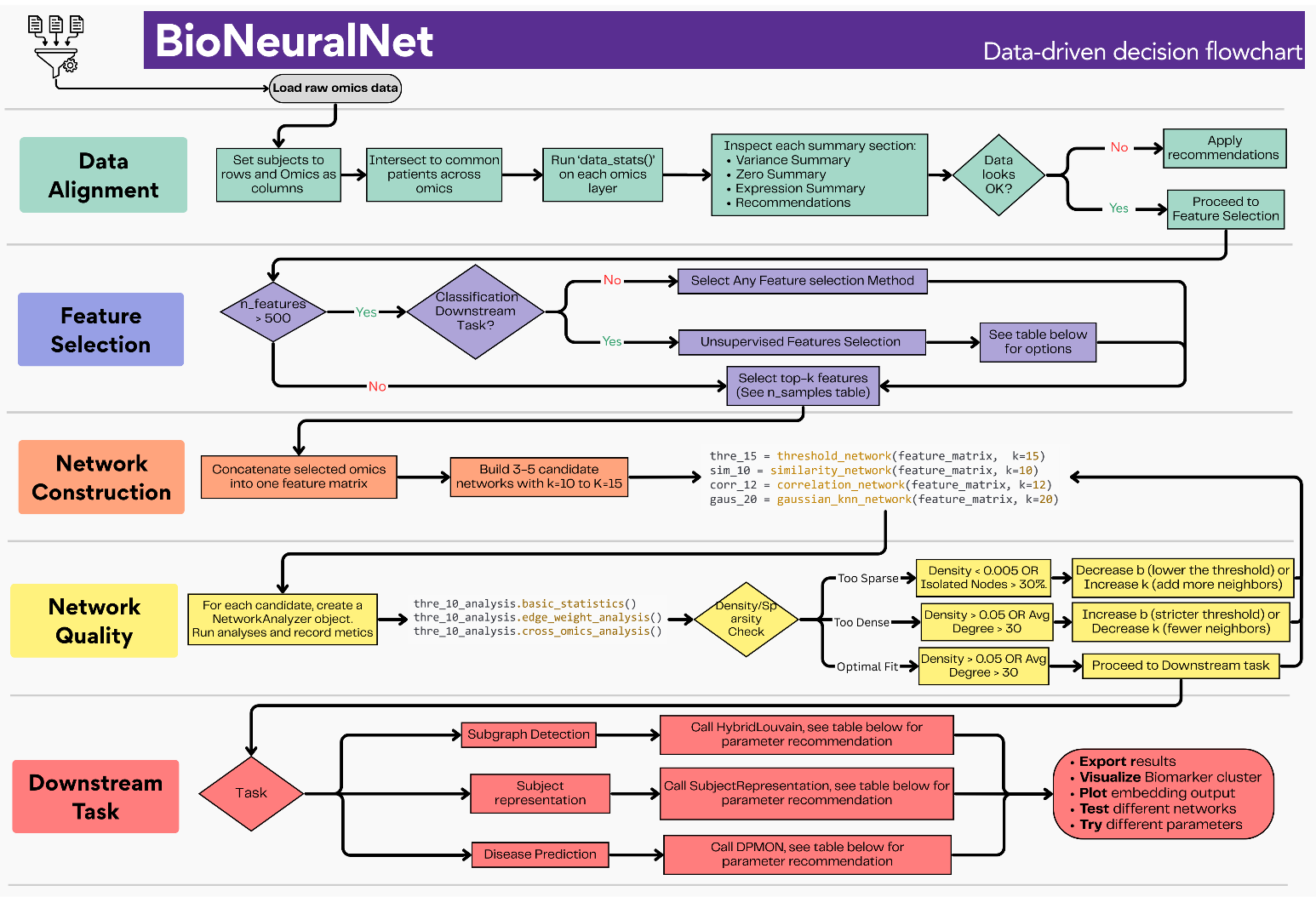
Network Calibration: After building candidate networks, the *NetworkAnalyzer* class provides metrics (Density, Isolated Nodes, Hubs) to help you decide if the topology is "Optimal," "Too Sparse," or "Too Dense" for a GNN architecture.

**1.3 Pipeline Parameter Recipes**

The recipes provided in the following tables are anchored in the empirical results derived from the TCGA workflows presented in the manuscript. These guidelines serve as a heuristic for calibrating the BioNeuralNet pipeline across diverse multi-omics contexts.

**Stage 1: Data Alignment & Quality Assessment**

Based on the output of the *data_stats()* diagnostic tool, use these rules to prepare data before feature selection.

| Condition | Action | Empirical Result (BRCA Data) |
| --- | --- | --- |
| Expression Bounded (0.0 to 1.0) | Run *m_transform*(eps=1e-7) to normalize data for network construction. | Flagged: Methylation (Min 0.012, Max 0.980) successfully converted to M-values (-6.3 to 5.6). |
| Missing Features (≥20%) | Run *sparse_filter*(missing_fraction=0.20) to remove features where missingness exceeds the imputation limit (e.g., hsa_mir_3654 at 66.19%). | Flagged: miRNA had 13.7% global NaNs; filter successfully removed 138 features exceeding the 20% feature-specific threshold. |
| High Exact Zeros | Apply log2(x+1) transformation to mitigate sparsity issues. | Passed: Zero mean was 0.000 across layers; data was already normalized upstream. |

**Stage 2: Feature Selection**

BioNeuralNet supports several options for dimensionality reduction to mitigate the high dimensionality of omics layers prior to network construction and embedding. The framework implements both unsupervised and supervised target strategies. For a complete list of feature selection methods, please visit: <https://bioneuralnet.readthedocs.io/en/latest/utils.html#feature-selection>

| Method | Selection Logic | Parameters & Functional Rules |
| --- | --- | --- |
| Variance Threshold | High-dimensional data (Number of omics > 10,000). | Fast pre-filter; retain top $k$ features (e.g., 5,000 for RNA/Meth). |
| Laplacian Score | Preserving patient clusters. | Set $n\_keep$to specify number of features to keep. |
| MAD Filter | Heavy-tailed distributions. | Use Median Absolute Deviation for outlier-heavy data. |
| Correlation Filter | Reducing signal redundancy. | Drop features with correlation > 0.85 to others. |

**Stage 3: Network Construction & Topology Selection**

Graph structure significantly impacts GNN performance. While BioNeuralNet supports various construction methods, GNNs favor regularized graphs with 0% isolated nodes to ensure stable message passing.

| Graph Type | Core Measure | Key Parameters | Sparsification & Options | Notes & Best Use Case |
| --- | --- | --- | --- | --- |
| Gaussian KNN | Gaussian (RBF) similarity on Euclidean distances | k, sigma (optional) | kNN per node; optional mutual edges, self-loops | Nonlinear Structures: Creates smooth similarity landscapes. sigma controls the "locality" (how fast similarity decays). |
| Similarity | Cosine similarity or Gaussian on Euclidean distance | k, metric (cosine, Euclidean) | kNN per node or global cutoff; optional mutual edges | High-Dimensional Data: Cosine is preferred for text/embeddings where vector angle matters more than magnitude. |
| Correlation | Pearson or Spearman correlation mapped to [0,1] | k (or threshold), correlation method | Per-node kNN or global cutoff; optional mutual edges | Statistical Trends: Captures linear (Pearson) or rank-based monotonic (Spearman) relationships between features. |
| Threshold | Soft-threshold absolute Pearson correlation | b (power), k | kNN on soft-threshold similarities; optional mutual edges | Gene Co-expression: The power parameter b suppresses weak noise to approximate a "scale-free" network topology. |

**1.4 Automated Hyperparameter Tuning (DPMON)**

While the DPMON module offers extensive configurability, BioNeuralNet is designed to eliminate manual guesswork and mitigate suboptimal configurations. Users can initialize DPMON with their respective multi-omics data, clinical variables, and network structures, and easily automate the optimization process by setting tune=True (which defaults to 20 tune_trials).

To ensure comprehensive optimization while avoiding over-parameterization, the automated tuner searches the following empirically validated space:

- GNN Layers (gnn_layer_num): 2, 3, or 4
- GNN Hidden Dimensions (gnn_hidden_dim): 32 or 64
- Learning Rate (lr): Log-uniform distribution (1e-4 to 8e-4)
- Weight Decay (weight_decay): Log-uniform distribution (1e-5 to 5e-3)
- Autoencoder Encoding Dimension (ae_encoding_dim): 4 or 8
- Feed-Forward Hidden Dimensions:
  - Layer 1 (nn_hidden_dim1): 128 or 256
  - Layer 2 (nn_hidden_dim2): 64

A complete framework and parameter recipes for decision making when using BioNeuralNet is available at: <https://bioneuralnet.readthedocs.io/en/latest/quick_start/data_framework.html>

**2. Methodological Details**

**2.1 Shared Preprocessing Pipeline**

All three cohorts follow an identical preprocessing pipeline. Raw multi-omics (mRNA, miRNA, DNA methylation) and clinical data were acquired from the Broad Institute FireHose pipeline. TCGA-BRCA PAM50 subtype labels were additionally obtained via TCGAbiolinks

**Omics Preprocessing (all cohorts):**

| Step | Details |
| --- | --- |
| Transpose | Raw files transposed from features x samples to samples x features |
| Barcode standardization | Patient barcodes trimmed to 12-character TCGA identifiers |
| Duplicate features | Averaged across aliquots |
| Methylation transform | Beta values converted to M-values via log2 transform |
| Column standardization | Special characters replaced, duplicate separators removed |
| Patient alignment | Intersection of patients present across all modalities and clinical data |

**2.2 Cohort-specific preprocessing differences:**

Depending on the missingness and sparsity inherent to each cancer cohort, specific filtering and imputation strategies were applied

| Step | TCGA-BRCA | TCGA-LGG | TCGA-KIPAN |
| --- | --- | --- | --- |
| Sparse feature filter (>20% missing) | Yes | No | No |
| Imputation method | KNN (k=5) | Mean imputation | Mean imputation |

**2.3 Feature Selection (all cohorts):**

To address high dimensionality and isolate the most informative variables, unsupervised feature selection was performed across all cohorts using Laplacian Score filtering. This method systematically evaluates each feature based on its ability to preserve the local manifold structure of the data. By emphasizing features that exhibit smoothness, meaning they vary minimally between closely related samples.

The Laplacian Score ($L_{r}$) for the $r$-th feature is calculated using the following objective function:

$$L_{r} = \frac{\sum_{ij} \left( x_{ri} - x_{rj} \right)^{2} W_{ij}}{\text{Var}\left( x_{r} \right)}$$

**Variable Definitions:**

- $L_{r}$: The computed Laplacian Score for feature r. Because the numerator calculates the penalty for variation between connected neighbors, lower scores indicate higher feature importance.
- $x_{ri}$ and $x_{rj}$: The standardized values of the $r$-th feature for sample $i$ and sample$j$, respectively. Prior to scoring, all feature vectors undergo standard scaling (Z-score normalization).
- $W_{ij}$: The edge weight between sample $i$ and sample $j$ in the global affinity network. This is derived by constructing a symmetric k-nearest neighbors connectivity graph. If sample $i$ and sample $j$ are neighbors, $W_{ij}$ = 1; otherwise, it is 0.
- $\text{Var}\left( x_{r} \right):$ The variance of feature r, weighted by the degree matrix of the graph. This denominator serves as a scale-invariant normalization factor, ensuring that the score reflects the local spatial variance relative to the global variance of the feature.

By filtering for the lowest Laplacian Scores, the following optimal subsets of features were retained per cohort to maximize computational efficiency while preserving biological signals:

| Modality | Features Retained |
| --- | --- |
| DNA Methylation | 400 |
| mRNA | 200 |
| miRNA | 100 |

See our online documentation for a comprehensive list of additional feature selection methods.

**2.4 Algorithmic Frameworks**

The following flowcharts and respective papers detail the internal mathematical and algorithmic workflows executed by BioNeuralNet downstream task modules.


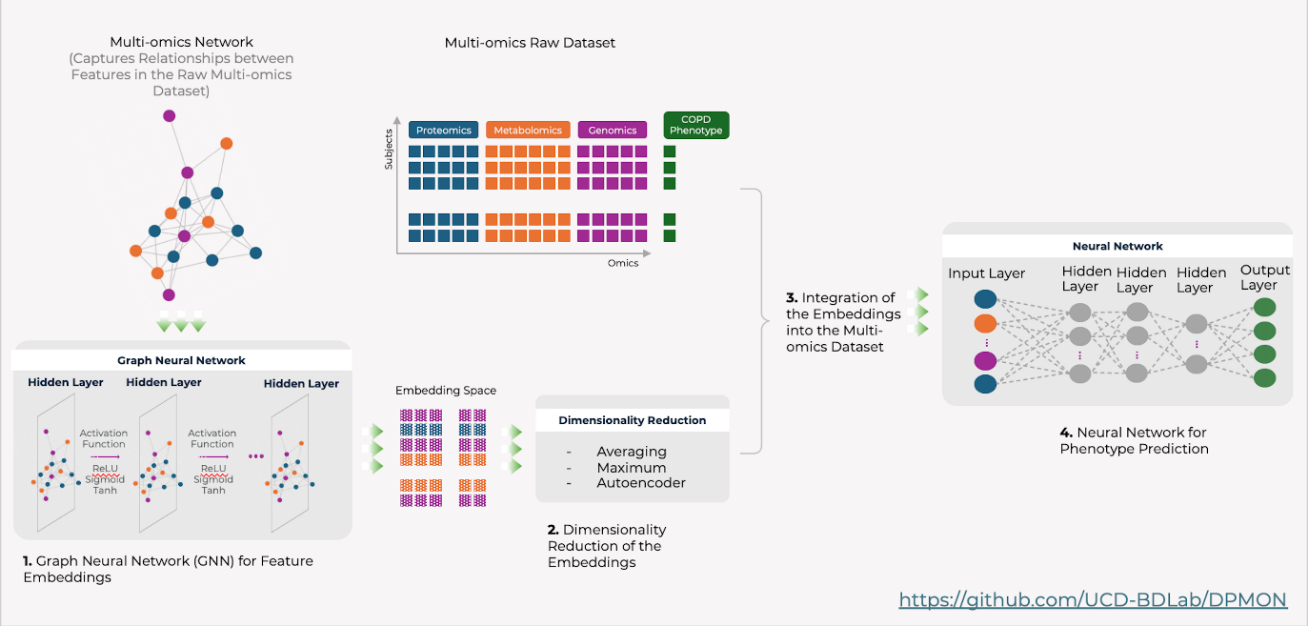


Algorithm 1. Uses enriched network embeddings and fusion approach to learn from multi-omics networks for disease prediction (Hussein et al., 2024). <https://doi.org/10.1109/BIBM62325.2024.10822233>


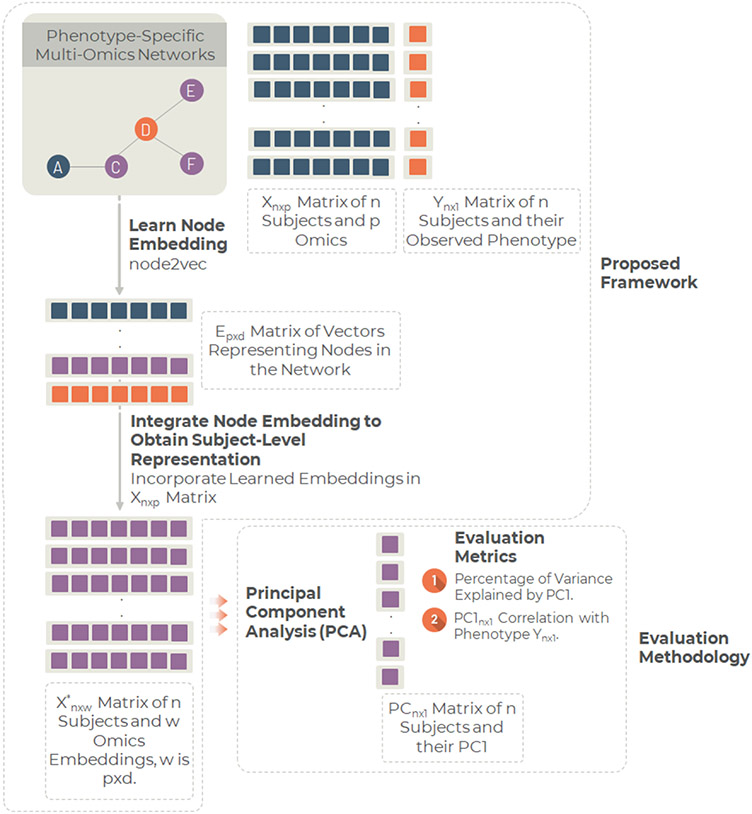


Algorithm 2*.* Generates subject representations by applying graph embedding techniques to multi-omics disease networks (Hussein et al., 2022). <https://doi.org/10.1109/BIBM55620.2022.9995707>


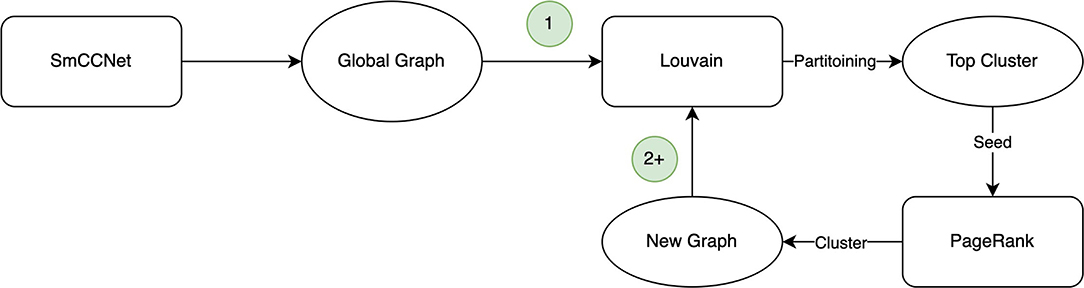


Algorithm 3. Focuses on significant subgraph detection within multi-omics networks to identify disease pathways (Abdel-Hafiz et al., 2022). <https://doi.org/10.3389/fdata.2022.894632>

**3. Additional Case Studies**

To demonstrate the versatility and predictive power of the BioNeuralNet framework, we evaluated its performance across multiple independent multi-omics cohorts.

**3.1 S1**: TCGA-BRCA - PAM50 Cancer Subtype Classification

**Cohort Summary:**

| Stage | Methylation | mRNA | miRNA | Clinical | PAM50 |
| --- | --- | --- | --- | --- | --- |
| Raw (features x samples) | 20,107 x 885 | 18,321 x 1,212 | 503 x 1,189 | 1,098 x 18 | 1,087 x 1 |
| Final aligned (samples x features) | 769 x 20,106 | 769 x 16,757 | 769 x 354 | 769 x 17 | 769 x 1 |
| After feature selection | 769 x 400 | 769 x 200 | 769 x 100 | 769 x 17 | 769 x 1 |

**Target Definition: PAM50 Subtype (5-class):**

LumA (n=419), LumB (n=140), Basal (n=130), Her2 (n=46), and Normal (n=34).

**Network Construction & Disease Prediction:** Two soft threshold co-expression networks were constructed. Absolute Pearson correlations between features were raised to power ($b$) and sparsified using a k-nearest neighbors mask ($k$). End-to-end phenotype prediction was performed using BioNeuralNet DPMON module, pairing the clinical and omics matrices with the following networks.

| Network | Function | Parameters | GNN Pairing |
| --- | --- | --- | --- |
| Threshold | threshold_network | $b$=6.3, $k$=22 | GCN |
| Threshold | threshold_network | $b$=6.7, $k$=15 | GAT |

**Network Quality Assessment**: Prior to downstream modeling, BioNeuralNet *NetworkAnalyzer* class was used to assess graph topology. Adjusting parameters ($b$,$k$) within the same network construction method yielded distinct structural topologies, varying in total edges and global density.

| Network Configuration | Nodes | Edges | Density | Isolated Nodes | Top Edge Weight |
| --- | --- | --- | --- | --- | --- |
| Threshold ($b$=6.3, $k$=22) | 700 | 7,700 | 0.0315 | 0 (0.0%) | 0.522 |
| Threshold ($b$=6.7, $k$=15) | 700 | 5,250 | 0.0215 | 0 (0.0%) | 0.616 |

The *NetworkAnalyzer* class also enables hub analysis, cross-omics connectivity, and clustering diagnostics for rapid structural review. For details: <https://bioneuralnet.readthedocs.io/en/latest/network.html#network-quality-assessment>

**Table S1: TCGA-BRCA Classification Results**

| Model | Accuracy | F1 Weighted | F1 Macro | Recall | Precision | AUC | AUPR |
| --- | --- | --- | --- | --- | --- | --- | --- |
| Logistic Regression | 0.834 +/- 0.017 | 0.828 +/- 0.018 | 0.749 +/- 0.040 | 0.735 +/- 0.050 | 0.786 +/- 0.041 | 0.955 +/- 0.014 | 0.818 +/- 0.036 |
| GCN | 0.832 +/- 0.030 | 0.829 +/- 0.029 | 0.744 +/- 0.062 | 0.742 +/- 0.068 | 0.779 +/- 0.069 | 0.949 +/- 0.016 | 0.814 +/- 0.049 |
| GAT | 0.829 +/- 0.030 | 0.824 +/- 0.029 | 0.743 +/- 0.060 | 0.735 +/- 0.051 | 0.784 +/- 0.077 | 0.946 +/- 0.017 | 0.811 +/- 0.044 |
| XGBoost | 0.828 +/- 0.034 | 0.816 +/- 0.034 | 0.739 +/- 0.041 | 0.714 +/- 0.035 | 0.805 +/- 0.069 | 0.955 +/- 0.011 | 0.826 +/- 0.034 |
| SVM | 0.817 +/- 0.025 | 0.812 +/- 0.025 | 0.731 +/- 0.039 | 0.721 +/- 0.043 | 0.761 +/- 0.040 | 0.953 +/- 0.016 | 0.812 +/- 0.036 |
| Random Forest | 0.804 +/- 0.018 | 0.781 +/- 0.016 | 0.668 +/- 0.047 | 0.635 +/- 0.040 | 0.786 +/- 0.077 | 0.949 +/- 0.008 | 0.796 +/- 0.023 |
| MLP | 0.804 +/- 0.027 | 0.796 +/- 0.026 | 0.677 +/- 0.044 | 0.672 +/- 0.043 | 0.711 +/- 0.051 | 0.939 +/- 0.014 | 0.759 +/- 0.049 |
| Decision Tree | 0.761 +/- 0.044 | 0.757 +/- 0.038 | 0.650 +/- 0.033 | 0.645 +/- 0.040 | 0.674 +/- 0.038 | 0.822 +/- 0.040 | 0.593 +/- 0.052 |

**3.2 S2:** TCGA-LGG (Lower Grade Glioma) Binary Survival Prediction

**Cohort Summary:**

| Stage | Methylation | mRNA | miRNA | Clinical |
| --- | --- | --- | --- | --- |
| Raw (features x samples) | 20,115 x 685 | 18,328 x 701 | 548 x 531 | 14 x 1,110 |
| Final aligned (samples x features) | 511 x 20,114 | 511 x 18328 | 511 x 548 | 511 x 13 |
| After feature selection | 511 x 400 | 511 x 200 | 511 x 100 | 511 x 13 |

**Target Definition:** Binary vital status, Alive (n=386) vs. Deceased (n=125).

**Network Construction & Disease Prediction:** End-to-end phenotype prediction was performed using the DPMON module, pairing the constructed networks with GNN architectures.

| Network | Function | Parameters | GNN Pairing |
| --- | --- | --- | --- |
| Cosine similarity | similarity_network | $metric$=”cosine”, $k$=22 | GCN |
| Spearman correlation | correlation_network | $method$=”spearman”, $k$=12 | GAT |

**Network Quality Assessment**: Prior to downstream modeling, BioNeuralNet *NetworkAnalyzer* class was used to assess graph topology. Different network construction methods and neighborhood constraints ($k$=22 vs. $k$=12) yields distinct structural topologies.

| Network Configuration | Nodes | Edges | Density | Isolated Nodes | Top Edge Weight |
| --- | --- | --- | --- | --- | --- |
| Cosine Similarity ($k$=22) | 700 | 7,700 | 0.0315 | 0 (0.0%) | 0.057 |
| Spearman Correlation $(k$=12) | 700 | 4,200 | 0.0172 | 0 (0.0%) | 0.095 |

The *NetworkAnalyzer* class also enables hub analysis, cross-omics connectivity, and clustering diagnostics for rapid structural review. For example, calling *NetworkAnalyzer.find_strongest_edges(5)* on the spearman_12 network generates the following table:

| Rank | Feature 1 | Feature 2 | Weight |
| --- | --- | --- | --- |
| 1 | hsa_mir_128_2 | hsa_mir_128_1 | 0.094614 |
| 2 | hsa_mir_23a | hsa_mir_27a | 0.094176 |
| 3 | hsa_mir_129_1 | hsa_mir_129_2 | 0.091661 |
| 4 | LMBRD2_92255 | LMTK2_22853 | 0.090525 |
| 5 | SAMD8_142891 | MAN1A2_10905 | 0.090381 |

Visit the online documentation for detailed code examples.

**Table S2:** TCGA-LGG Results

| Model | Accuracy | F1 Weighted | F1 Macro | Recall | Precision | AUC | AUPR |
| --- | --- | --- | --- | --- | --- | --- | --- |
| GCN | 0.773 +/- 0.024 | **0.780 +/- 0.022** | **0.714 +/- 0.030** | **0.739 +/- 0.050** | **0.710 +/- 0.025** | 0.798 +/- 0.038 | 0.549 +/- 0.069 |
| GAT | **0.781 +/- 0.030** | 0.776 +/- 0.039 | 0.694 +/- 0.067 | 0.698 +/- 0.073 | 0.703 +/- 0.054 | **0.802 +/- 0.042** | **0.579 +/- 0.063** |
| Logistic Regression | 0.771 +/- 0.011 | 0.753 +/- 0.011 | 0.646 +/- 0.022 | 0.635 +/- 0.024 | 0.685 +/- 0.023 | 0.785 +/- 0.035 | 0.551 +/- 0.051 |
| Random Forest | 0.780 +/- 0.023 | 0.756 +/- 0.030 | 0.644 +/- 0.051 | 0.631 +/- 0.041 | 0.699 +/- 0.051 | 0.727 +/- 0.034 | 0.516 +/- 0.061 |
| SVM | 0.748 +/- 0.023 | 0.729 +/- 0.022 | 0.613 +/- 0.035 | 0.606 +/- 0.030 | 0.642 +/- 0.036 | 0.756 +/- 0.024 | 0.507 +/- 0.032 |
| XGBoost | 0.767 +/- 0.038 | 0.742 +/- 0.041 | 0.624 +/- 0.061 | 0.613 +/- 0.052 | 0.676 +/- 0.067 | 0.756 +/- 0.031 | 0.523 +/- 0.041 |
| MLP | 0.736 +/- 0.031 | 0.728 +/- 0.025 | 0.625 +/- 0.036 | 0.625 +/- 0.042 | 0.641 +/- 0.032 | 0.721 +/- 0.041 | 0.477 +/- 0.033 |
| Decision Tree | 0.699 +/- 0.055 | 0.693 +/- 0.038 | 0.581 +/- 0.036 | 0.584 +/- 0.032 | 0.599 +/- 0.041 | 0.585 +/- 0.034 | 0.339 +/- 0.040 |

**S2.1: Additional Demonstrative Workflows**

As a secondary demonstration of the framework’s utility, we leveraged BioNeuralNet native functionality to perform adjacent workflows using the LGG cohort as a case study:

- Latent Space Visualization (Figure S2.1): We projected feature embeddings from the DPMON module into a 2D latent space. This allows users to inspect how different omics layers cluster and evaluate the distribution of learned representations within the model.
- Phenotype-Driven Subgraph Detection (Table S2.2): Using HybridLouvain module, we iteratively pruned the global Spearman network. This process systematically isolates biologically meaningful subgraphs that maximize correlation with patient vital status.
- Network Topology & Hub Analysis (Figure S2.2 / Table S2.3): We extracted a representative 22-node module (Iteration 4) to showcase the framework’s plotting utilities and its ability to identify central molecular features (e.g., HIVEP3, DBH, and ERMP1).

**Figure S2.1:** 2D latent space to visualization of DPMON embeddings.


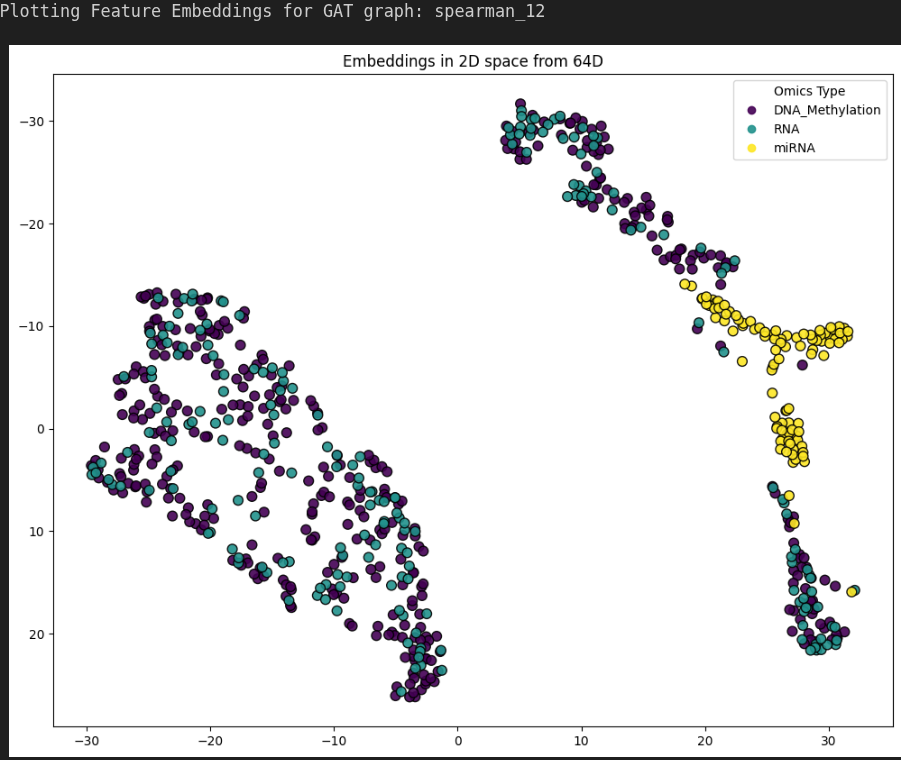


**Table S2.2:** HybridLouvain subnetwork identification.

| Iteration | Nodes Remaining | Correlation | Conductance |
| --- | --- | --- | --- |
| 0 | 402 | 0.3061 | 0.0004 |
| 1 | 214 | 0.3252 | 0.1177 |
| 2 | 93 | 0.3305 | 0.3262 |
| 3 | 46 | 0.3365 | 0.3093 |
| **4** | **22** | **0.3363** | **0.3877** |
| 5 | 10 | 0.3487 | 0.4134 |
| 6 | 5 | 0.3568 | 0.4013 |

**Figure S2.2:** Visualization of Subnetwork found by HybridLouvain. Iteration 4 from Table S2.2.

**
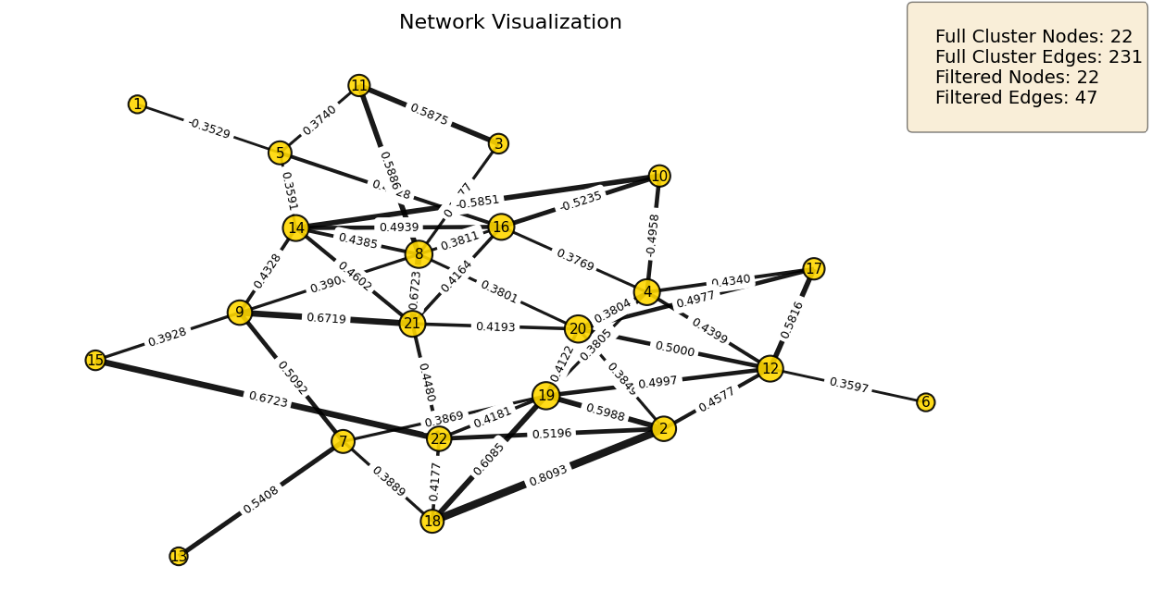
**

**Table S2.3:** Reference Table for Subnetwork shown above. Figure S2.2

| Index | Omic Feature | Degree |
| --- | --- | --- |
| 20 | HIVEP3 | 7 |
| 19 | DBH | 7 |
| 8 | ERMP1 | 7 |
| 12 | LFNG | 6 |
| 21 | MIR23A | 6 |
| 4 | THADA | 6 |
| 16 | JAKMIP3 | 6 |
| 14 | DLGAP4 | 6 |
| 2 | HRH2 | 5 |
| 22 | CLIC1 | 5 |

**3.3 S3:** TCGA-KIPAN - Binary Cancer Staging

**Cohort Summary:**

| Stage | Methylation | mRNA | miRNA | Clinical |
| --- | --- | --- | --- | --- |
| Raw (features x samples) | 20,117 x 867 | 18,272 x 1,020 | 472 x 1,005 | 20 x 941 |
| Final Aligned (samples x features) | 658 x 20,116 | 658 x 18,272 | 658 x 472 | 658 x 19 |
| After feature selection | 633 x 400 | 633 x 200 | 633 x 100 | 633 x 19 |

**Target Definition:**

Binary cancer stage: Early (Stages I/II, n=417) vs. Late (Stages III/IV, n=216).

**Network Construction & Disease Prediction:** End-to-end phenotype prediction was performed using the DPMON module, pairing the constructed networks with GNN architectures.

| Network | Function | Parameters | GNN Pairing |
| --- | --- | --- | --- |
| Spearman correlation | correlation_network | $method$=”spearman”, $k$=12 | SAGE |
| Cosine Similarity | similarity_network | $metric$=”cosine”, $k$=22 | SAGE |

**Network Quality Assessment**: Prior to downstream modeling, BioNeuralNet *NetworkAnalyzer* class was used to assess graph topology. Different network construction methods and neighborhood constraints ($k$=22 vs. $k$=12) yields distinct structural topologies.

| Network Configuration | Nodes | Edges | Density | Isolated Nodes | Top Edge Weight |
| --- | --- | --- | --- | --- | --- |
| Cosine Similarity ($k$=22) | 700 | 7,700 | 0.0315 | 0 (0.0%) | 0.057 |
| Spearman Correlation $(k$=12) | 700 | 4,200 | 0.0172 | 0 (0.0%) | 0.095 |

The *NetworkAnalyzer* class also enables hub analysis, cross-omics connectivity, and clustering diagnostics for rapid structural review. For example, calling *NetworkAnalyzer.find_strongest_edges(5)* on the spearman_12 network generates the following table:

| Rank | Feature 1 | Feature 2 | Weight |
| --- | --- | --- | --- |
| 1 | hsa_mir_29b_2 | hsa_mir_29b_1 | 0.11075 |
| 2 | hsa_mir_141 | hsa_mir_200c | 0.103668 |
| 3 | hsa_mir_194_2 | hsa_mir_194_1 | 0.100373 |
| 4 | hsa_mir_365_1 | hsa_mir_365_2 | 0.100198 |
| 5 | hsa_mir_19b_2 | hsa_mir_19a | 0.099948 |

Visit the online documentation for detailed code examples.

**Table S3:** TCGA-KIPAN Results

| Model | Accuracy | F1 Weighted | F1 Macro | Recall | Precision | AUC | AUPR |
| --- | --- | --- | --- | --- | --- | --- | --- |
| SAGE | 0.762 +/- 0.025 | 0.763 +/- 0.025 | 0.738 +/- 0.027 | 0.741 +/- 0.029 | 0.738 +/- 0.027 | 0.800 +/- 0.015 | 0.693 +/- 0.029 |
| Logistic Regression | 0.756 +/- 0.045 | 0.749 +/- 0.046 | 0.715 +/- 0.052 | 0.708 +/- 0.049 | 0.734 +/- 0.055 | 0.798 +/- 0.035 | 0.690 +/- 0.049 |
| XGBoost | 0.757 +/- 0.030 | 0.750 +/- 0.032 | 0.716 +/- 0.038 | 0.708 +/- 0.037 | 0.732 +/- 0.036 | 0.791 +/- 0.026 | 0.663 +/- 0.046 |
| SVM | 0.758 +/- 0.049 | 0.750 +/- 0.049 | 0.715 +/- 0.055 | 0.707 +/- 0.052 | 0.739 +/- 0.058 | 0.788 +/- 0.030 | 0.667 +/- 0.047 |
| Random Forest | 0.755 +/- 0.028 | 0.737 +/- 0.035 | 0.694 +/- 0.043 | 0.683 +/- 0.038 | 0.744 +/- 0.039 | 0.799 +/- 0.018 | 0.680 +/- 0.029 |
| MLP | 0.746 +/- 0.032 | 0.740 +/- 0.031 | 0.706 +/- 0.033 | 0.700 +/- 0.031 | 0.720 +/- 0.036 | 0.790 +/- 0.024 | 0.645 +/- 0.039 |
| Decision Tree | 0.674 +/- 0.032 | 0.674 +/- 0.032 | 0.641 +/- 0.039 | 0.649 +/- 0.045 | 0.647 +/- 0.038 | 0.664 +/- 0.058 | 0.472 +/- 0.052 |

**Table S3.1:** TCGA-KIPAN network-GNN paring comparison

| Model | Graph | Accuracy | F1 Weighted | F1 Macro | Recall | Precision | AUC | AUPR |
| --- | --- | --- | --- | --- | --- | --- | --- | --- |
| **GAT** | pearson_12 | 0.746 +/- 0.032 | 0.748 +/- 0.029 | 0.723 +/- 0.029 | 0.729 +/- 0.028 | 0.723 +/- 0.029 | 0.781 +/- 0.025 | 0.673 +/- 0.042 |
| **GAT** | similarity_22 | 0.755 +/- 0.034 | 0.755 +/- 0.032 | 0.729 +/- 0.035 | 0.731 +/- 0.036 | 0.732 +/- 0.036 | 0.798 +/- 0.030 | 0.675 +/- 0.056 |
| **GCN** | pearson_12 | 0.746 +/- 0.027 | 0.747 +/- 0.026 | 0.720 +/- 0.030 | 0.722 +/- 0.033 | 0.721 +/- 0.030 | 0.783 +/- 0.028 | 0.668 +/- 0.041 |
| **GCN** | similarity_22 | 0.744 +/- 0.044 | 0.745 +/- 0.040 | 0.719 +/- 0.041 | 0.722 +/- 0.037 | 0.722 +/- 0.043 | 0.778 +/- 0.031 | 0.668 +/- 0.052 |
| **SAGE** | pearson_12 | 0.762 +/- 0.025 | 0.763 +/- 0.025 | 0.738 +/- 0.027 | 0.741 +/- 0.029 | 0.738 +/- 0.027 | 0.800 +/- 0.015 | 0.693 +/- 0.029 |
| **SAGE** | similarity_22 | 0.758 +/- 0.046 | 0.758 +/- 0.045 | 0.731 +/- 0.049 | 0.732 +/- 0.049 | 0.732 +/- 0.050 | 0.780 +/- 0.030 | 0.671 +/- 0.055 |

**3.4 S4:** ROSMAP (Alzheimer's Disease) Biomarker Discovery

**Cohort Summary:**

| Stage | Methylation | mRNA | miRNA | Clinical |
| --- | --- | --- | --- | --- |
| Raw (features x samples) | 420,132 x 741 | 55,889 x 642 | 309 x 704 | 18 x 3584 |
| Data Aligned (samples x features) | 521 x 420,132 | 521 x 55,889 | 521 x 309 | 521 x 17 |
| Variance top 100k (Methylation only) | 521 x 100,000 | - | - | - |
| Anova-F & Random Forest Intersection k=8000 | 521 x 772 | 521 x 2054 | - | - |
| After feature selection | 521 x 772 | 521 x 2054 | 521 x 309 | 521 x 17 |
| Final feature selection (Variance k=300) | 521 x 300 | 521 x 300 | 521 x 300 | 521 x 17 |

**Target Definition:** Multi-class cognitive diagnosis derived from clinical cogdx scores.

No Cognitive Impairment (NCI, n=168), Mild Cognitive Impairment (MCI, n=134), and Dementia (n=219).

**Network Construction & Embedding Generation**: Multi-omic networks were constructed and processed using the DPMON module with default parameters to generate latent feature embeddings, without performing downstream phenotype prediction.

| Network | Function | Parameters | GNN Pairing |
| --- | --- | --- | --- |
| Cosine Similarity | similarity_network | $metric$=”cosine”, $k$=10 | GAT (default) |

**Subnetwork Discovery & Hub Analysis:**

Application of the Hybrid Louvain clustering module identified five distinct subnetworks. Graph Attention Network (GAT) embeddings were generated for the largest module to visualize the topological structure of the latent space. The smaller, highly dense subnetworks were mapped as discrete interaction graphs to identify specific regulatory hubs and high-degree drivers of disease pathology.

**Figure S4.1:** Embedding space 900 Multi-omic Network. GAT separation across omics types forming a biological hierarchy.

The following clusters represent subnetworks of highly correlated omics features, visualized using force-directed graphs to aid interpretation. The weight threshold is an adjustable parameter used to filter weak edges for visual clarity. Biologically, these modules reveal distinct mechanisms of Alzheimer's pathology, collectively mapping the multi-layered breakdown of the AD brain [1-8,15].

- 50 node subnetwork: Highlights synaptic plasticity and cytoskeletal scaffolding (driven by MYO10 and HOMER3).
- 25 node subnetwork: Captures white matter degeneration and cellular senescence (OLIG1, FOXO4).
- 12 node subnetwork: Identifies a critical failure in metabolic cofactor recycling and aberrant cell cycle re-entry (QDPR, CDK18).
- 6 node subnetwork: Points toward protein clearance and lysosomal dysfunction.

**Table S4.1** HybridLouvain subnetwork identification

| Iteration | Nodes Remaining | Correlation |
| --- | --- | --- |
| 0 | 600 | 0.0857 |
| 1 | 50 | 0.1563 |
| 2 | 25 | 0.1608 |
| 3 | 12 | 0.1554 |
| 4 | 6 | 0.1921 |

**Figure S4.1** Embedding space 900 Multi-omic Network.


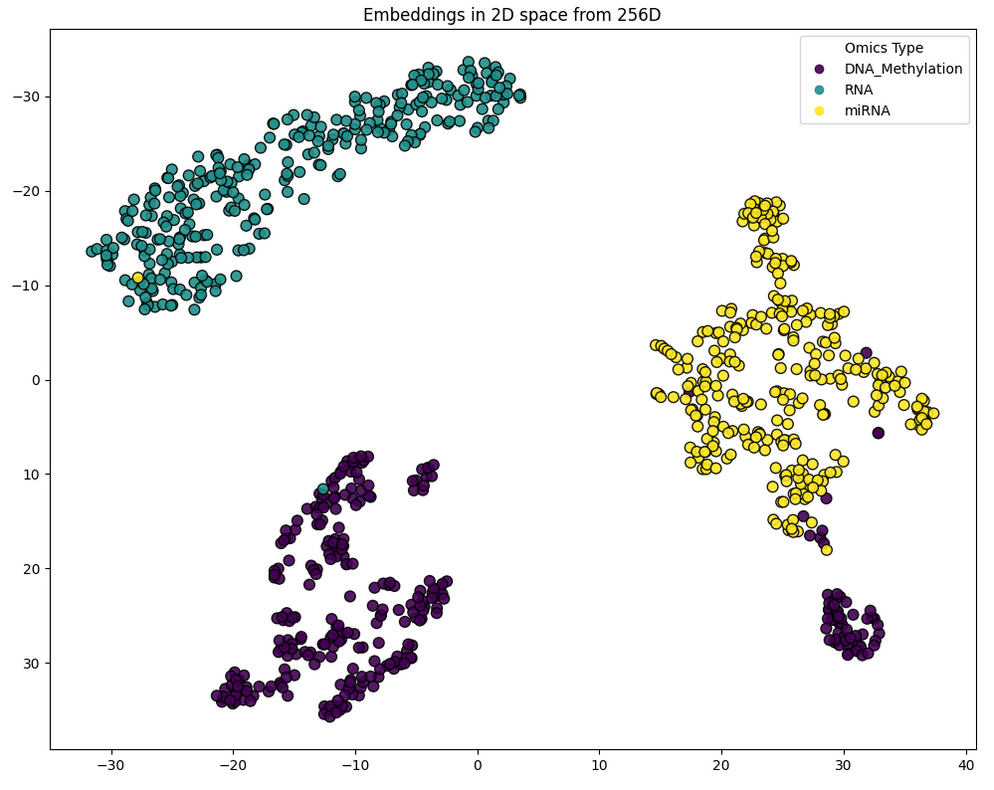


**Figure S4.2** Network visualization of the 50-node "NCI vs Rest" submodule filtered at a 0.7 weight threshold.


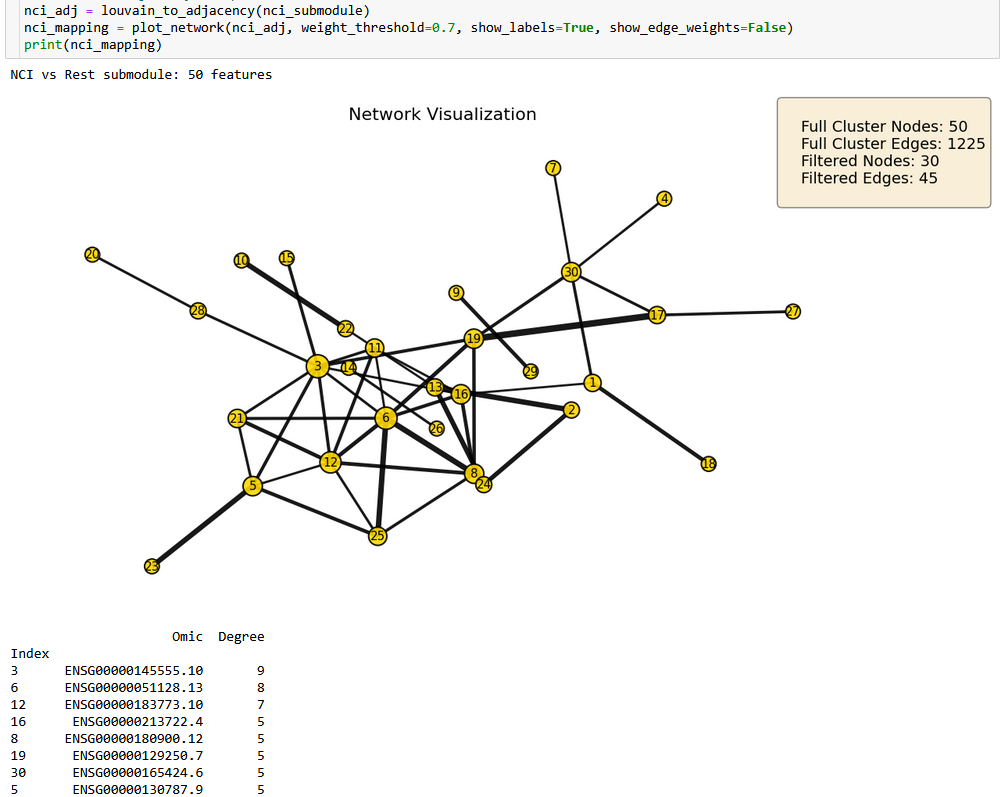


**Figure S4.3**. Network visualization of the 25-node "NCI vs Rest" subgraph filtered at a 0.6 weight threshold.


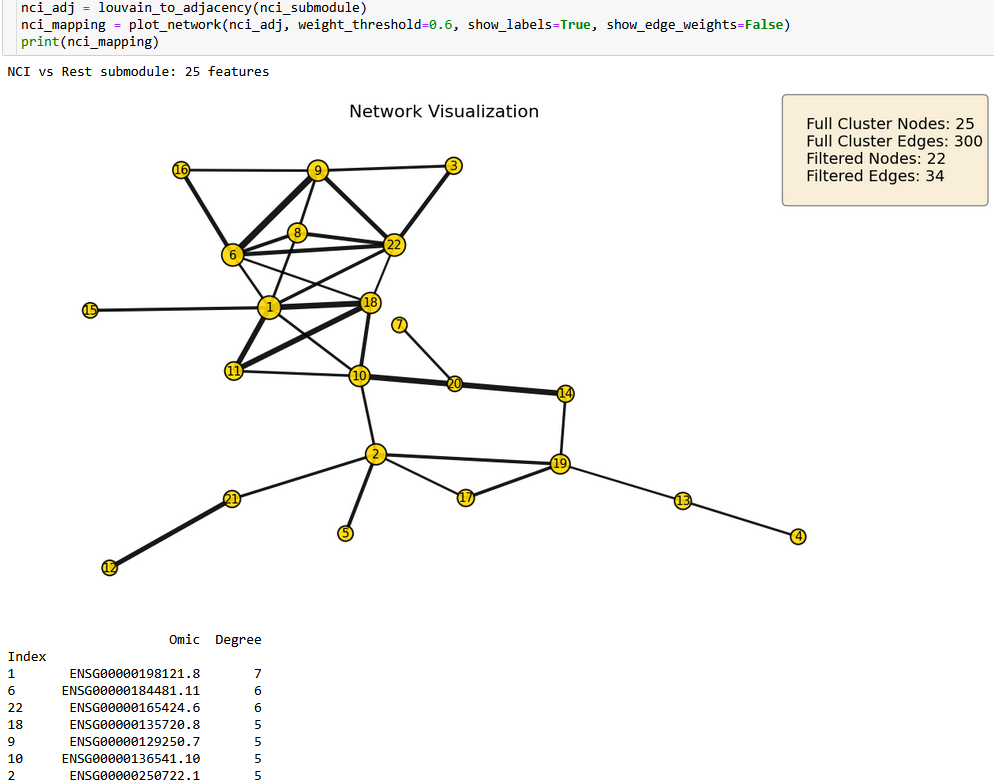


**Figure S4.4**. Network visualization of the 12-node "NCI vs Rest" subgraph filtered at a 0.45 weight threshold.


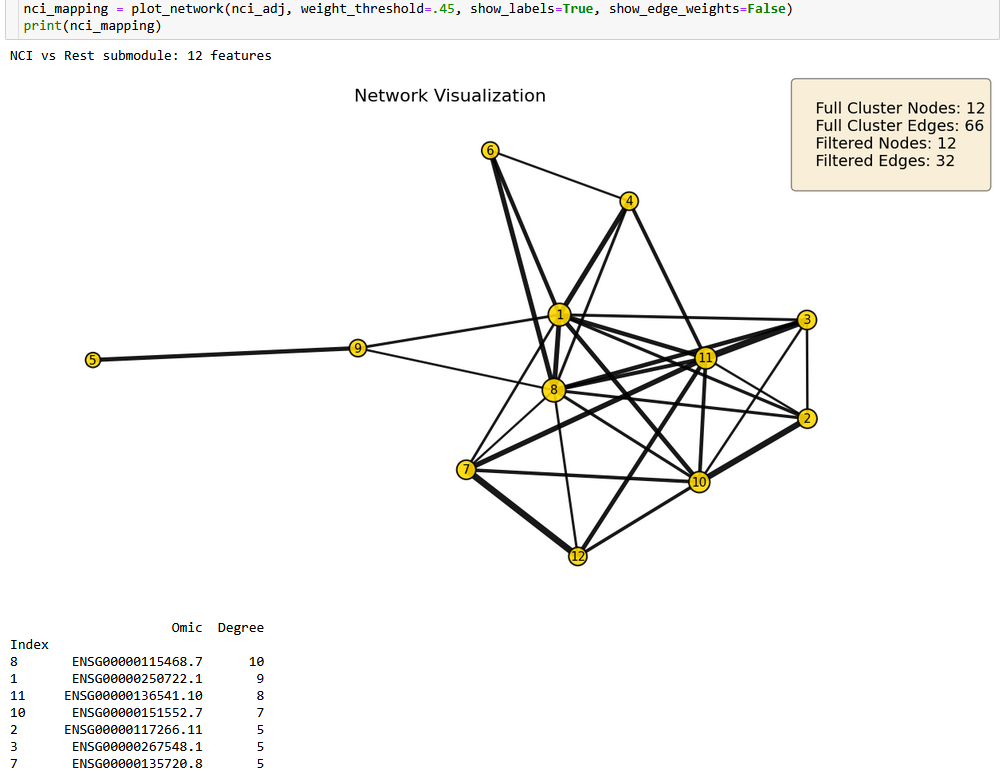


**Figure S4.5** Network visualization of the 6-node "NCI vs Rest" subgraph filtered at a 0-weight threshold.


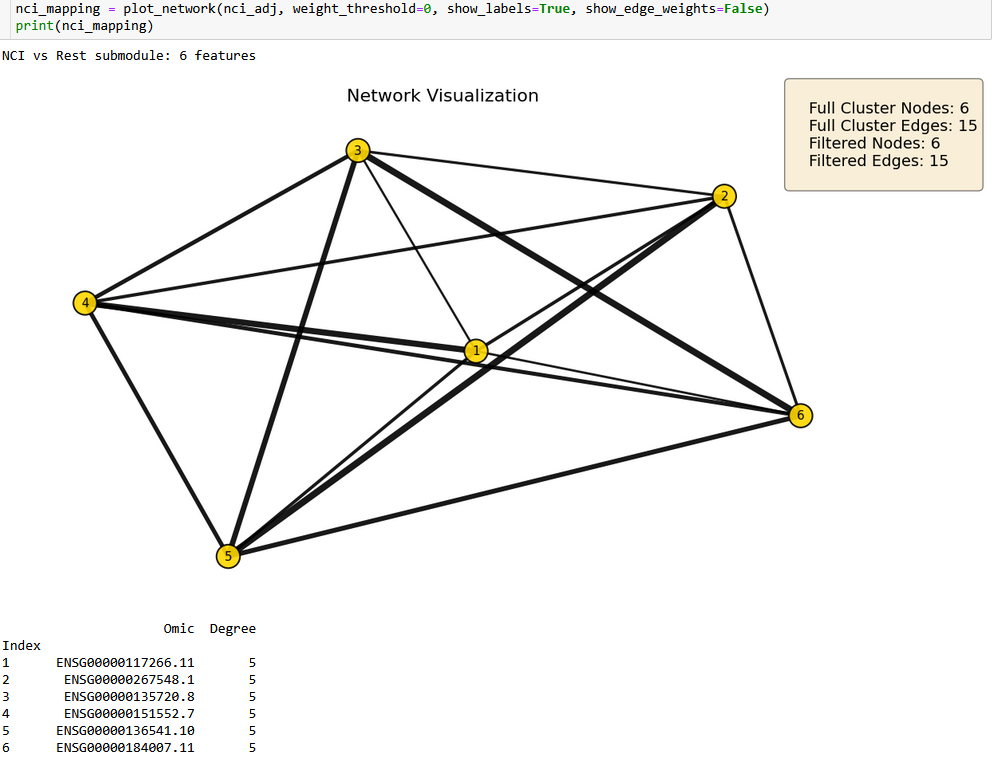


**References**

1. Baar MP, Brandt RM, Putavet DA, Klein JD, Derks KW, Bourgeois BR, et al. Targeted apoptosis of senescent cells restores tissue homeostasis in response to chemotoxicity and aging. Cell. 2017;169(1):132–147.

2. Foong J, Parry SW, McNulty S. The role of tetrahydrobiopterin (BH4) metabolism in Alzheimer's disease. J Neurochem. 2020;152(S1):125–139.

3. Herrup K, Yang Y. Cell cycle regulation in the postmitotic neuron: oxymoron or new biology? Nat Rev Neurosci. 2007;8(5):368–378.

4. Herskovits AZ, Davies P. The regulation of tau phosphorylation by PCTAIRE 3: implications for the pathogenesis of Alzheimer's disease. Neurobiol Dis. 2006;23(2):398–408.

5. Mathys H, Davila-Velderrain J, Peng Z, Gao F, Mohammadi S, Young JZ, et al. Single-cell transcriptomic analysis of Alzheimer’s disease. Nature. 2019;570(7761):332–337.

6. Nixon RA. The role of autophagy in neurodegenerative disease. Nat Med. 2013;19(8):983–997.

7. Parisiadou L, Bethani I, Michaki V, Krousti K, Rapti G, Efthimiopoulos S. Homer2 and Homer3 interact with amyloid precursor protein and inhibit Aβ production. Neurobiol Dis. 2008;30(3):353–364.

8. Penzes P, Vanleeuwen JE. Impaired regulation of dendritic spines in Alzheimer's disease. Alzheimers Res Ther. 2011;3(6):35.

9. Davis CF, Ricketts CJ, Wang M, Yang L, Cherniack AD, Shen H, et al. The somatic genomic landscape of chromophobe renal cell carcinoma. Cancer Cell. 2014;26(3):319–330.

10. Frew IJ, Moch H. A clearer view of the molecular complexity of clear cell renal cell carcinoma. Annu Rev Pathol. 2015;10:263–289.

11. Picard N, et al. Dmrt2 and Hmx2 direct intercalated cell diversity in the mammalian kidney through antagonistic and supporting regulatory processes. Proc Natl Acad Sci U S A. 2025;122(20). doi:10.1073/pnas.2418471122.

12. Schrader AJ, Rauer-Müller JM, Valadez-Banet S, Kuczyk MA, Hofmann R. SLC26A7 is a diagnostic marker of chromophobe renal cell carcinoma. Urol Oncol. 2016;34(10):486.e1.

13. Uhlén M, Fagerberg L, Hallström BM, Lindskog C, Oksvold P, Mardinoglu A, et al. Tissue-based map of the human proteome. Science. 2015;347(6220):1260419.

14. Vergho D, Kneitz S, Rosenwald A, Schartl M, Spahn M, Riedmiller H, et al. Combination of expression of CK7 and CLCN5 and CLCNK as a specific marker for the diagnosis of chromophobe renal cell carcinoma. Urology. 2012;79(2):484.e1.

15. AD Knowledge Portal. Available from: https://adknowledgeportal.org/. Data generated from postmortem brain tissue provided by the Religious Orders Study and Rush Memory and Aging Project (ROSMAP) cohort at Rush Alzheimer’s Disease Center, Rush University Medical Center, Chicago. Supported by Cure Alzheimer’s Fund and NIH grants AG058002, AG062377, NS110453, NS115064, AG062335, AG074003, NS127187, MH119509, HG008155, RF1AG062377, RF1AG054321, R01AG054012, and GM087237.
