# Supplementary material for: BioNeuralNet: a graph neural network based Multi-Omics network data analysis tool
Source: Bioinformatics. 2026 Jun 10;42(6):btag365. doi: 10.1093/bioinformatics/btag365 (PMC13293062; doi:10.1093/bioinformatics/btag365)
Supplement: btag365_Supplementary_Data [file btag365_supplementary_data.zip › BioNeuralNet_Supplementary.pdf]

BioNeuralNet Supplementary Materials

Table of Contents

1. Internal Architecture

- 1.1 Package Overview
- 1.2 Data Decision Framework & Navigation Guide
- 1.3 Pipeline Parameter Recipes
- 1.4 Automated Hyperparameter Tuning

2. Methodological Details

- 2.1 Shared Preprocessing Pipeline
- 2.2 Cohort-Specific Preprocessing Differences
- 2.3 Feature Selection and Laplacian Score
- 2.4 Algorithmic Frameworks

3. Additional Case Studies

- 3.1 S1: TCGA-BRCA (Breast Cancer) Subtype Classification
- 3.2 S2: TCGA-LGG (Lower Grade Glioma) Survival Prediction
- 3.3 S3: TCGA-KIPAN (Pan-Kidney) Cancer Staging
- 3.4 S4: ROSMAP (Alzheimer's Disease) Biomarker Discovery

1.1 Package Overview

BioNeuralNet is an open-source, modular Python framework tailored for end-to-end network-based multi-omics data analysis. The package architecture is decoupled into functional submodules that mirror the stages of the analytical workflow.

- *datasets*: Manages ingestion via the *DatasetLoader* and provides built-in access to benchmark cohorts including TCGA-BRCA, LGG, KIPAN and synthetic data for experimentation.
- *utils*: Contains several diagnostic and preprocessing tools for data alignment, missing value imputation, and Beta-to-M transformations.
- *feature_selection*: Implements dimensionality reduction strategies such as Laplacian Score, MAD, and Variance Thresholding.
- *network*: Facilitates the construction of multi-omics networks using Similarity, Correlation, and Gaussian KNN strategies.
- *network_embedding*: Generates low-dimensional representations using Graph Neural Network (GNN) architectures including GCN, GAT, SAGE, and GIN.
- *clustering*: Identifies disease-associated subgraphs through hybrid community detection algorithms like Hybrid Louvain and Correlated PageRank.
- *downstream_task*: Executes specialized pipelines including DPMON for phenotype prediction and Subject Representation for patient-level profiling.
- *metrics* & *external_tools*: Provides topological evaluation, visualization utilities, and interoperability with R-based exports.

1.2 Data Decision Framework & Navigation Guide

Multi-omics datasets are notoriously high-dimensional, sparse, and noisy. To ensure the pipeline remains robust across different experimental contexts, we provide a structured Data-Driven Decision Flowchart (see image below). This guide serves as a heuristic for navigating the mathematical trade-offs inherent in multi-omics analysis, such as avoiding GNN over-smoothing or network fragmentation.

Key Decision Heuristics:

- Preprocessing Quality: Use the data_stats() summary to determine if missingness or bounding issues require specific imputation or transformation actions.
- Feature Scaling: Unsupervised selection (e.g., Laplacian Score) is recommended for high-dimensional layers to isolate biological signal from noise before network construction.
- Network Calibration: After building candidate networks, the *NetworkAnalyzer* class provides metrics (Density, Isolated Nodes, Hubs) to help you decide if the topology is "Optimal," "Too Sparse," or "Too Dense" for a GNN architecture.

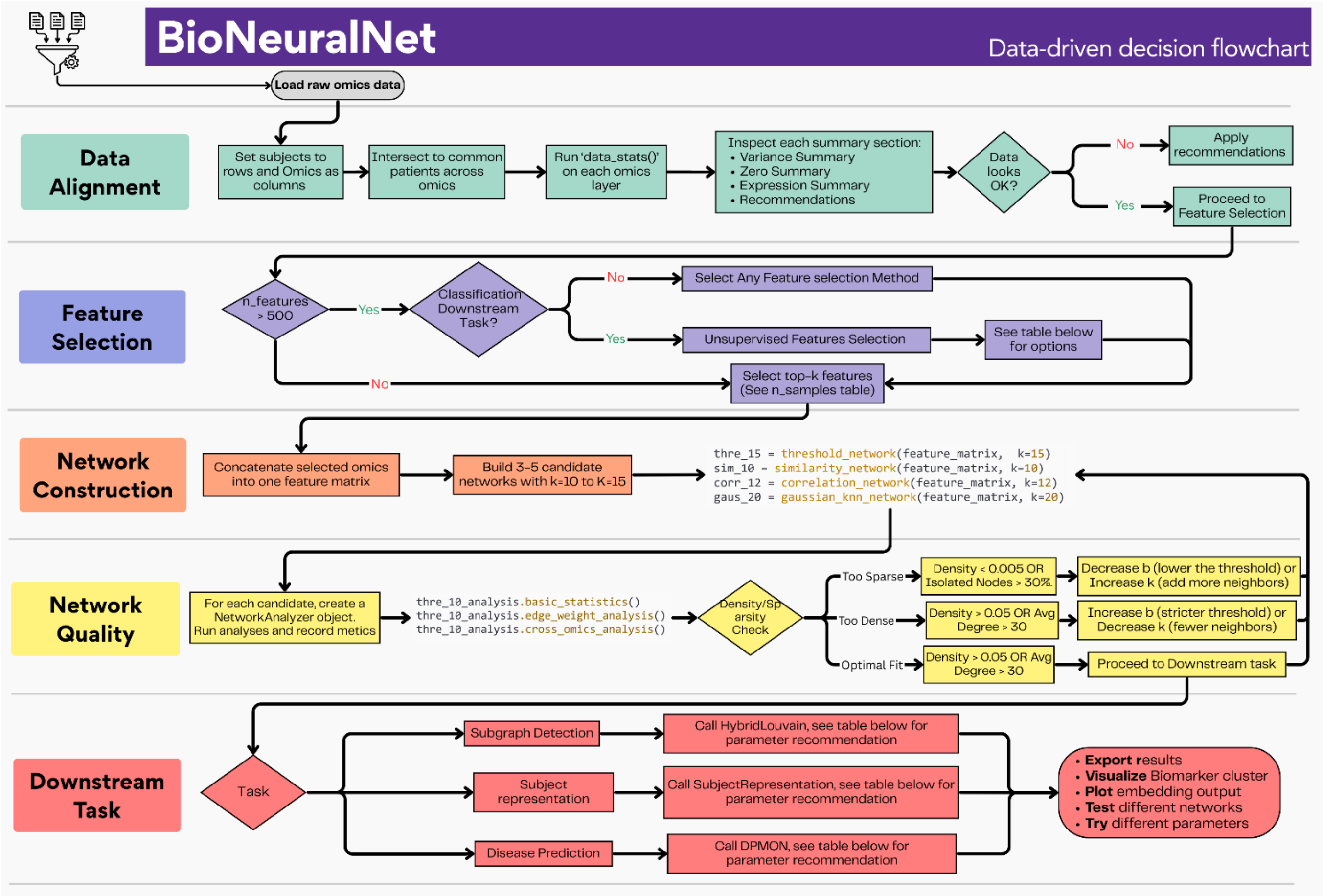

1.3 Pipeline Parameter Recipes

The recipes provided in the following tables are anchored in the empirical results derived from the TCGA workflows presented in the manuscript. These guidelines serve as a heuristic for calibrating the BioNeuralNet pipeline across diverse multi-omics contexts.

Stage 1: Data Alignment & Quality Assessment

Based on the output of the *data_stats()* diagnostic tool, use these rules to prepare data before feature selection.

Condition	Action	Empirical Result (BRCA Data)
Expression Bounded (0.0 to 1.0)	Run *m_transform*(eps=1e-7) to normalize data for network construction.	Flagged: Methylation (Min 0.012, Max 0.980) successfully converted to M-values (-6.3 to 5.6).
Missing Features (≥20%)	Run *sparse_filter*(missing_fraction=0.20) to remove features where missingness exceeds the imputation limit (e.g., hsa_mir_3654 at 66.19%).	Flagged: miRNA had 13.7% global NaNs; filter successfully removed 138 features exceeding the 20% feature-specific threshold.
High Exact Zeros	Apply log2(x+1) transformation to mitigate sparsity issues.	Passed: Zero mean was 0.000 across layers; data was already normalized upstream.

Stage 2: Feature Selection

BioNeuralNet supports several options for dimensionality reduction to mitigate the high dimensionality of omics layers prior to network construction and embedding. The framework implements both unsupervised and supervised target strategies. For a complete list of feature selection methods, please visit: https://bioneuralnet.readthedocs.io/en/latest/utils.html#feature-selection

Method	Selection Logic	Parameters & Functional Rules
Variance Threshold	High-dimensional data (Number of omics > 10,000).	Fast pre-filter; retain top k features (e.g., 5,000 for RNA/Meth).
Laplacian Score	Preserving patient clusters.	Set *n_keep* to specify number of features to keep.
MAD Filter	Heavy-tailed distributions.	Use Median Absolute Deviation for outlier-heavy data.
Correlation Filter	Reducing signal redundancy.	Drop features with correlation > 0.85 to others.

Stage 3: Network Construction & Topology Selection

Graph structure significantly impacts GNN performance. While BioNeuralNet supports various construction methods, GNNs favor regularized graphs with 0% isolated nodes to ensure stable message passing.

Graph Type	Core Measure	Key Parameters	Sparsification & Options	Notes & Best Use Case
Gaussian KNN	Gaussian (RBF) similarity on Euclidean distances	k, sigma (optional)	kNN per node; optional mutual edges, self-loops	Nonlinear Structures: Creates smooth similarity landscapes. sigma controls the "locality" (how fast similarity decays).
Similarity	Cosine similarity or Gaussian on Euclidean distance	k, metric (cosine, Euclidean)	kNN per node or global cutoff; optional mutual edges	High-Dimensional Data: Cosine is preferred for text/embeddings where vector angle matters more than magnitude.
Correlation	Pearson or Spearman correlation mapped to [0,1]	k (or threshold), correlation method	Per-node kNN or global cutoff; optional mutual edges	Statistical Trends: Captures linear (Pearson) or rank-based monotonic (Spearman) relationships between features.
Threshold	Soft-threshold absolute Pearson correlation	b (power), k	kNN on soft-threshold similarities; optional mutual edges	Gene Co-expression: The power parameter b suppresses weak noise to approximate a "scale-free" network topology.

1.4 Automated Hyperparameter Tuning (DPMON)

While the DPMON module offers extensive configurability, BioNeuralNet is designed to eliminate manual guesswork and mitigate suboptimal configurations. Users can initialize DPMON with their respective multi-omics data, clinical variables, and network structures, and easily automate the optimization process by setting tune=True (which defaults to 20 tune_trials).

To ensure comprehensive optimization while avoiding over-parameterization, the automated tuner searches the following empirically validated space:

- GNN Layers (gnn_layer_num): 2, 3, or 4
- GNN Hidden Dimensions (gnn_hidden_dim): 32 or 64
- Learning Rate (lr): Log-uniform distribution (1e-4 to 8e-4)
- Weight Decay (weight_decay): Log-uniform distribution (1e-5 to 5e-3)
- Autoencoder Encoding Dimension (ae_encoding_dim): 4 or 8
- Feed-Forward Hidden Dimensions:
    - Layer 1 (nn_hidden_dim1): 128 or 256
    - Layer 2 (nn_hidden_dim2): 64

A complete framework and parameter recipes for decision making when using BioNeuralNet is available at: https://bioneuralnet.readthedocs.io/en/latest/quick_start/data_framework.html

2. Methodological Details

2.1 Shared Preprocessing Pipeline

All three cohorts follow an identical preprocessing pipeline. Raw multi-omics (mRNA, miRNA, DNA methylation) and clinical data were acquired from the Broad Institute FireHose pipeline. TCGA-BRCA PAM50 subtype labels were additionally obtained via TCGAbiolinks

Omics Preprocessing (all cohorts):

Step	Details
Transpose	Raw files transposed from features x samples to samples x features
Barcode standardization	Patient barcodes trimmed to 12-character TCGA identifiers
Duplicate features	Averaged across aliquots
Methylation transform	Beta values converted to M-values via log2 transform
Column standardization	Special characters replaced, duplicate separators removed
Patient alignment	Intersection of patients present across all modalities and clinical data

2.2 Cohort-specific preprocessing differences:

Depending on the missingness and sparsity inherent to each cancer cohort, specific filtering and imputation strategies were applied

Step	TCGA-BRCA	TCGA-LGG	TCGA-KIPAN
Sparse feature filter (>20% missing)	Yes	No	No
Imputation method	KNN (k=5)	Mean imputation	Mean imputation

2.3 Feature Selection (all cohorts):

To address high dimensionality and isolate the most informative variables, unsupervised feature selection was performed across all cohorts using Laplacian Score filtering. This method systematically evaluates each feature based on its ability to preserve the local manifold structure of the data. By emphasizing features that exhibit smoothness, meaning they vary minimally between closely related samples.

The Laplacian Score (L_r) for the r-th feature is calculated using the following objective function:

$$L_r = \frac{\sum_{ij}\left(x_{ri} - x_{rj}\right)^2 W_{ij}}{\mathrm{Var}(x_r)}$$

Variable Definitions:

- L_r: The computed Laplacian Score for feature r. Because the numerator calculates the penalty for variation between connected neighbors, lower scores indicate higher feature importance.
- x_{ri} and x_{rj}: The standardized values of the r-th feature for sample i and sample j, respectively. Prior to scoring, all feature vectors undergo standard scaling (Z-score normalization).
- W_{ij}: The edge weight between sample i and sample j in the global affinity network. This is derived by constructing a symmetric k-nearest neighbors connectivity graph. If sample i and sample j are neighbors, W_{ij} = 1; otherwise, it is 0.
- $\mathrm{Var}(x_r)$: The variance of feature r, weighted by the degree matrix of the graph. This denominator serves as a scale-invariant normalization factor, ensuring that the score reflects the local spatial variance relative to the global variance of the feature.

By filtering for the lowest Laplacian Scores, the following optimal subsets of features were retained per cohort to maximize computational efficiency while preserving biological signals:

**Modality**	**Features Retained**
DNA Methylation	400
mRNA	200
miRNA	100

See our online documentation for a comprehensive list of additional feature selection methods.

2.4 Algorithmic Frameworks

The following flowcharts and respective papers detail the internal mathematical and algorithmic workflows executed by BioNeuralNet downstream task modules.

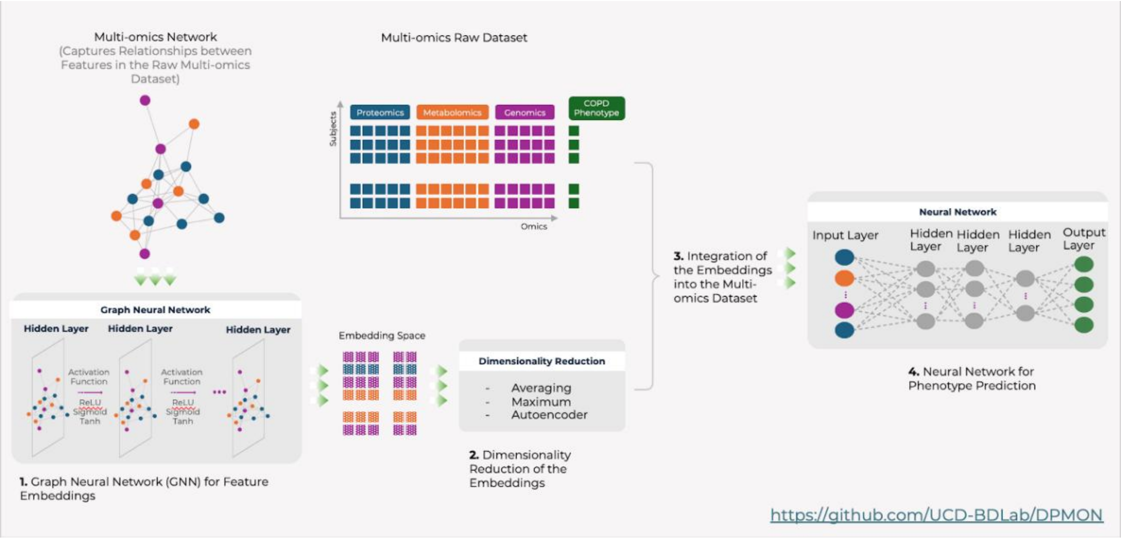

Algorithm 1. Uses enriched network embeddings and fusion approach to learn from multi-omics networks for disease prediction (Hussein et al., 2024). https://doi.org/10.1109/BIBM62325.2024.10822233

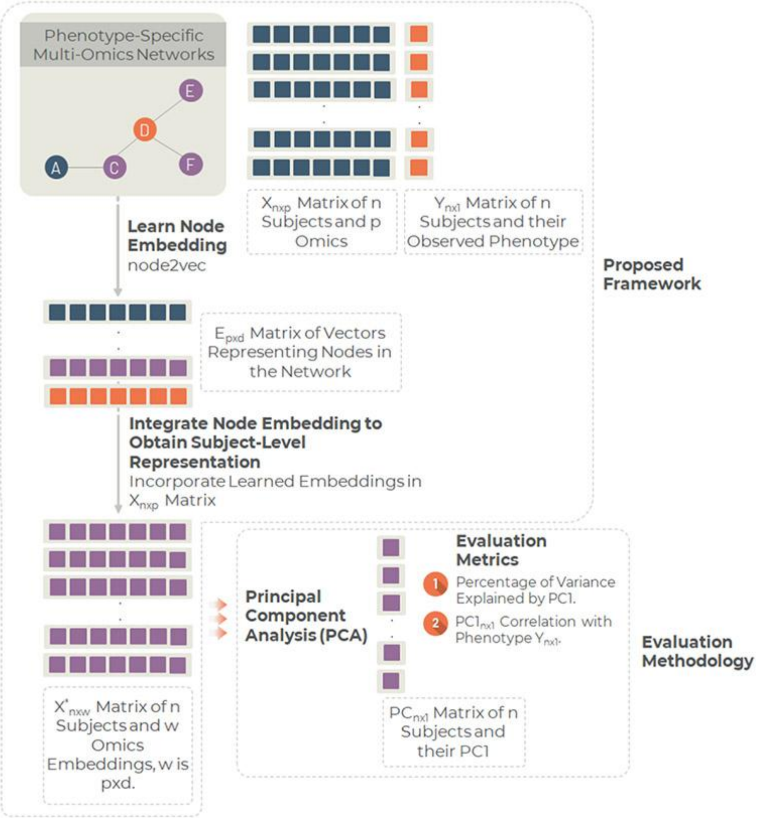

Algorithm 2. Generates subject representations by applying graph embedding techniques to multi-omics disease networks (Hussein et al., 2022). https://doi.org/10.1109/BIBM55620.2022.9995707

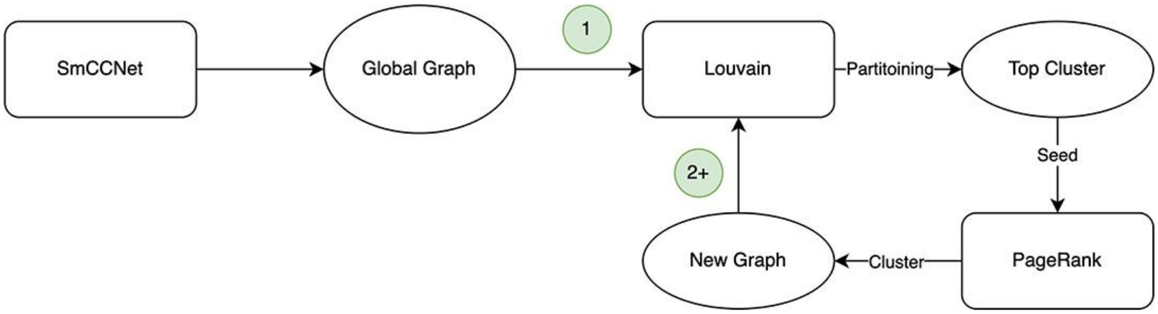

Algorithm 3. Focuses on significant subgraph detection within multi-omics networks to identify disease pathways (Abdel-Hafiz et al., 2022). https://doi.org/10.3389/fdata.2022.894632

3. Additional Case Studies

To demonstrate the versatility and predictive power of the BioNeuralNet framework, we evaluated its performance across multiple independent multi-omics cohorts.

3.1 S1: TCGA-BRCA - PAM50 Cancer Subtype Classification

Cohort Summary:

Stage	Methylation	mRNA	miRNA	Clinical	PAM50
Raw (features x samples)	20,107 x 885	18,321 x 1,212	503 x 1,189	1,098 x 18	1,087 x 1
Final aligned (samples x features)	769 x 20,106	769 x 16,757	769 x 354	769 x 17	769 x 1
After feature selection	769 x 400	769 x 200	769 x 100	769 x 17	769 x 1

Target Definition: PAM50 Subtype (5-class):

LumA (n=419), LumB (n=140), Basal (n=130), Her2 (n=46), and Normal (n=34).

Network Construction & Disease Prediction: Two soft threshold co-expression networks were constructed. Absolute Pearson correlations between features were raised to power (b) and sparsified using a k-nearest neighbors mask (k). End-to-end phenotype prediction was performed using BioNeuralNet DPMON module, pairing the clinical and omics matrices with the following networks.

Network	Function	Parameters	GNN Pairing
Threshold	threshold_network	b=6.3, k=22	GCN
Threshold	threshold_network	b=6.7, k=15	GAT

Network Quality Assessment: Prior to downstream modeling, BioNeuralNet *NetworkAnalyzer* class was used to assess graph topology. Adjusting parameters (b,k) within the same network construction method yielded distinct structural topologies, varying in total edges and global density.

Network Configuration	Nodes	Edges	Density	Isolated Nodes	Top Edge Weight
Threshold (b=6.3, k=22)	700	7,700	0.0315	0 (0.0%)	0.522
Threshold (b=6.7, k=15)	700	5,250	0.0215	0 (0.0%)	0.616

The *NetworkAnalyzer* class also enables hub analysis, cross-omics connectivity, and clustering diagnostics for rapid structural review. For details: https://bioneuralnet.readthedocs.io/en/latest/network.html#network-quality-assessment

Table S1: TCGA-BRCA Classification Results

Model	Accuracy	F1 Weighted	F1 Macro	Recall	Precision	AUC	AUPR
Logistic Regression	0.834 +/- 0.017	0.828 +/- 0.018	0.749 +/- 0.040	0.735 +/- 0.050	0.786 +/- 0.041	0.955 +/- 0.014	0.818 +/- 0.036
GCN	0.832 +/- 0.030	0.829 +/- 0.029	0.744 +/- 0.062	0.742 +/- 0.068	0.779 +/- 0.069	0.949 +/- 0.016	0.814 +/- 0.049
GAT	0.829 +/- 0.030	0.824 +/- 0.029	0.743 +/- 0.060	0.735 +/- 0.051	0.784 +/- 0.077	0.946 +/- 0.017	0.811 +/- 0.044
XGBoost	0.828 +/- 0.034	0.816 +/- 0.034	0.739 +/- 0.041	0.714 +/- 0.035	0.805 +/- 0.069	0.955 +/- 0.011	0.826 +/- 0.034
SVM	0.817 +/- 0.025	0.812 +/- 0.025	0.731 +/- 0.039	0.721 +/- 0.043	0.761 +/- 0.040	0.953 +/- 0.016	0.812 +/- 0.036
Random Forest	0.804 +/- 0.018	0.781 +/- 0.016	0.668 +/- 0.047	0.635 +/- 0.040	0.786 +/- 0.077	0.949 +/- 0.008	0.796 +/- 0.023
MLP	0.804 +/- 0.027	0.796 +/- 0.026	0.677 +/- 0.044	0.672 +/- 0.043	0.711 +/- 0.051	0.939 +/- 0.014	0.759 +/- 0.049
Decision Tree	0.761 +/- 0.044	0.757 +/- 0.038	0.650 +/- 0.033	0.645 +/- 0.040	0.674 +/- 0.038	0.822 +/- 0.040	0.593 +/- 0.052

3.2 S2: TCGA-LGG (Lower Grade Glioma) Binary Survival Prediction

Cohort Summary:

Stage	Methylation	mRNA	miRNA	Clinical
Raw (features x samples)	20,115 x 685	18,328 x 701	548 x 531	14 x 1,110
Final aligned (samples x features)	511 x 20,114	511 x 18328	511 x 548	511 x 13
After feature selection	511 x 400	511 x 200	511 x 100	511 x 13

Target Definition: Binary vital status, Alive (n=386) vs. Deceased (n=125).

Network Construction & Disease Prediction: End-to-end phenotype prediction was performed using the DPMON module, pairing the constructed networks with GNN architectures.

Network	Function	Parameters	GNN Pairing
Cosine similarity	similarity_network	*metric*=”cosine”, *k*=22	GCN
Spearman correlation	correlation_network	*method*=”spearman”, *k*=12	GAT

Network Quality Assessment: Prior to downstream modeling, BioNeuralNet *NetworkAnalyzer* class was used to assess graph topology. Different network construction methods and neighborhood constraints (*k*=22 vs. *k*=12) yields distinct structural topologies.

Network Configuration	Nodes	Edges	Density	Isolated Nodes	Top Edge Weight
Cosine Similarity (*k*=22)	700	7,700	0.0315	0 (0.0%)	0.057
Spearman Correlation (*k*=12)	700	4,200	0.0172	0 (0.0%)	0.095

The *NetworkAnalyzer* class also enables hub analysis, cross-omics connectivity, and clustering diagnostics for rapid structural review. For example, calling *NetworkAnalyzer.find_strongest_edges(5)* on the spearman_12 network generates the following table:

Rank	Feature 1	Feature 2	Weight
1	hsa_mir_128_2	hsa_mir_128_1	0.094614
2	hsa_mir_23a	hsa_mir_27a	0.094176
3	hsa_mir_129_1	hsa_mir_129_2	0.091661
4	LMBRD2_92255	LMTK2_22853	0.090525
5	SAMD8_142891	MAN1A2_10905	0.090381

Visit the online documentation for detailed code examples.

Table S2: TCGA-LGG Results

Model	Accuracy	F1 Weighted	F1 Macro	Recall	Precision	AUC	AUPR
GCN	0.773 +/- 0.024	**0.780 +/- 0.022**	**0.714 +/- 0.030**	**0.739 +/- 0.050**	**0.710 +/- 0.025**	0.798 +/- 0.038	0.549 +/- 0.069
GAT	**0.781 +/- 0.030**	0.776 +/- 0.039	0.694 +/- 0.067	0.698 +/- 0.073	0.703 +/- 0.054	**0.802 +/- 0.042**	**0.579 +/- 0.063**
Logistic Regression	0.771 +/- 0.011	0.753 +/- 0.011	0.646 +/- 0.022	0.635 +/- 0.024	0.685 +/- 0.023	0.785 +/- 0.035	0.551 +/- 0.051
Random Forest	0.780 +/- 0.023	0.756 +/- 0.030	0.644 +/- 0.051	0.631 +/- 0.041	0.699 +/- 0.051	0.727 +/- 0.034	0.516 +/- 0.061
SVM	0.748 +/- 0.023	0.729 +/- 0.022	0.613 +/- 0.035	0.606 +/- 0.030	0.642 +/- 0.036	0.756 +/- 0.024	0.507 +/- 0.032
XGBoost	0.767 +/- 0.038	0.742 +/- 0.041	0.624 +/- 0.061	0.613 +/- 0.052	0.676 +/- 0.067	0.756 +/- 0.031	0.523 +/- 0.041
MLP	0.736 +/- 0.031	0.728 +/- 0.025	0.625 +/- 0.036	0.625 +/- 0.042	0.641 +/- 0.032	0.721 +/- 0.041	0.477 +/- 0.033
Decision Tree	0.699 +/- 0.055	0.693 +/- 0.038	0.581 +/- 0.036	0.584 +/- 0.032	0.599 +/- 0.041	0.585 +/- 0.034	0.339 +/- 0.040

S2.1: Additional Demonstrative Workflows

As a secondary demonstration of the framework's utility, we leveraged BioNeuralNet native functionality to perform adjacent workflows using the LGG cohort as a case study:

- Latent Space Visualization (Figure S2.1): We projected feature embeddings from the DPMON module into a 2D latent space. This allows users to inspect how different omics layers cluster and evaluate the distribution of learned representations within the model.
- Phenotype-Driven Subgraph Detection (Table S2.2): Using HybridLouvain module, we iteratively pruned the global Spearman network. This process systematically isolates biologically meaningful subgraphs that maximize correlation with patient vital status.
- Network Topology & Hub Analysis (Figure S2.2 / Table S2.3): We extracted a representative 22-node module (Iteration 4) to showcase the framework's plotting utilities and its ability to identify central molecular features (e.g., HIVEP3, DBH, and ERMP1).

Figure S2.1: 2D latent space to visualization of DPMON embeddings.

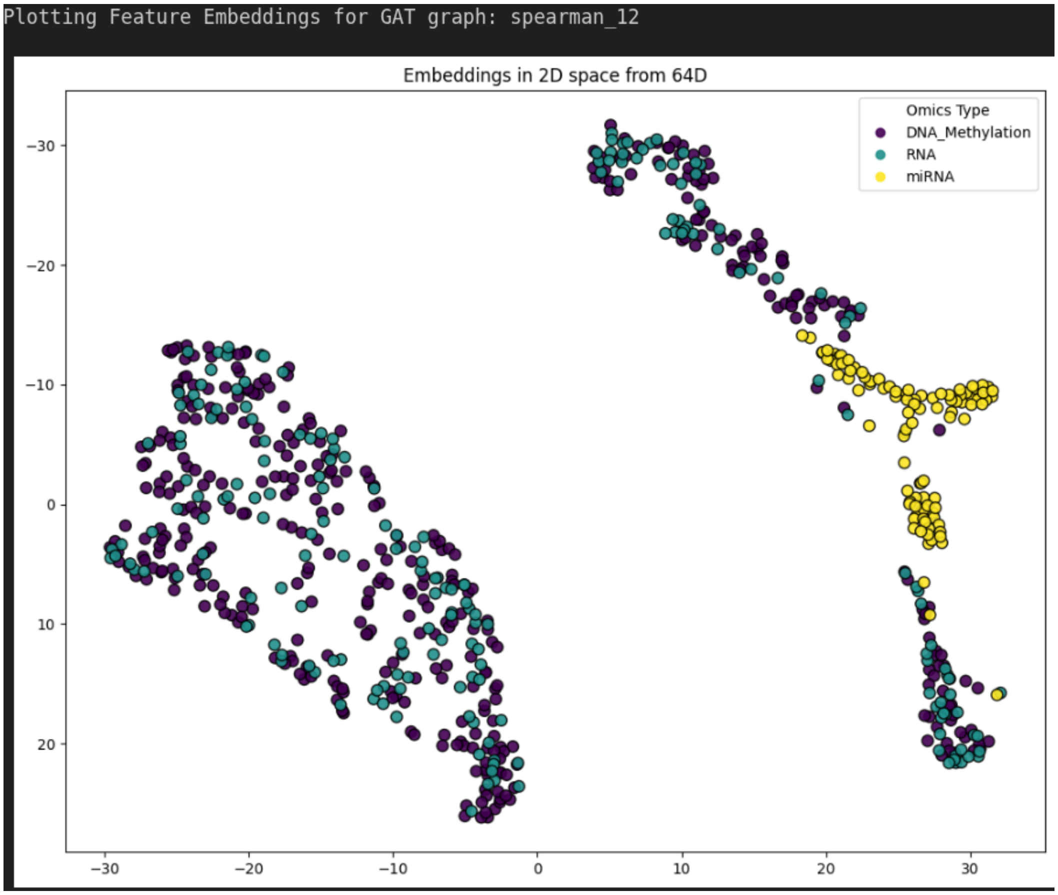

Table S2.2: HybridLouvain subnetwork identification.

Iteration	Nodes Remaining	Correlation	Conductance
0	402	0.3061	0.0004
1	214	0.3252	0.1177
2	93	0.3305	0.3262
3	46	0.3365	0.3093
**4**	**22**	**0.3363**	**0.3877**
5	10	0.3487	0.4134
6	5	0.3568	0.4013

Figure S2.2: Visualization of Subnetwork found by HybridLouvain. Iteration 4 from Table S2.2.

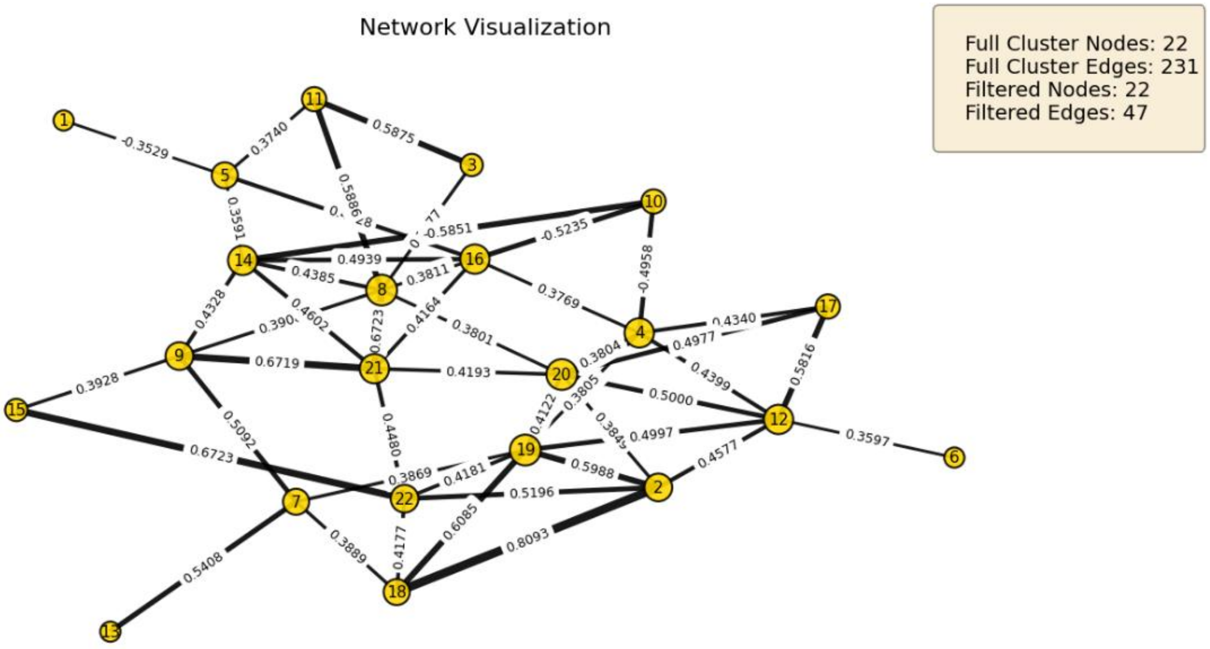

Table S2.3: Reference Table for Subnetwork shown above. Figure S2.2

Index	Omic Feature	Degree
20	HIVEP3	7
19	DBH	7
8	ERMP1	7
12	LFNG	6
21	MIR23A	6
4	THADA	6
16	JAKMIP3	6
14	DLGAP4	6
2	HRH2	5
22	CLIC1	5

3.3 S3: TCGA-KIPAN - Binary Cancer Staging

Cohort Summary:

Stage	Methylation	mRNA	miRNA	Clinical
Raw (features x samples)	20,117 x 867	18,272 x 1,020	472 x 1,005	20 x 941
Final Aligned (samples x features)	658 x 20,116	658 x 18,272	658 x 472	658 x 19
After feature selection	633 x 400	633 x 200	633 x 100	633 x 19

Target Definition:

Binary cancer stage: Early (Stages I/II, n=417) vs. Late (Stages III/IV, n=216).

Network Construction & Disease Prediction: End-to-end phenotype prediction was performed using the DPMON module, pairing the constructed networks with GNN architectures.

Network	Function	Parameters	GNN Pairing
Spearman correlation	correlation_network	*method*=”spearman”, k=12	SAGE
Cosine Similarity	similarity_network	*metric*=”cosine”, k=22	SAGE

Network Quality Assessment: Prior to downstream modeling, BioNeuralNet *NetworkAnalyzer* class was used to assess graph topology. Different network construction methods and neighborhood constraints (k=22 vs. k=12) yields distinct structural topologies.

Network Configuration	Nodes	Edges	Density	Isolated Nodes	Top Edge Weight
Cosine Similarity (k=22)	700	7,700	0.0315	0 (0.0%)	0.057
Spearman Correlation (k=12)	700	4,200	0.0172	0 (0.0%)	0.095

The *NetworkAnalyzer* class also enables hub analysis, cross-omics connectivity, and clustering diagnostics for rapid structural review. For example, calling *NetworkAnalyzer.find_strongest_edges(5)* on the spearman_12 network generates the following table:

Rank	Feature 1	Feature 2	Weight
1	hsa_mir_29b_2	hsa_mir_29b_1	0.11075
2	hsa_mir_141	hsa_mir_200c	0.103668
3	hsa_mir_194_2	hsa_mir_194_1	0.100373
4	hsa_mir_365_1	hsa_mir_365_2	0.100198
5	hsa_mir_19b_2	hsa_mir_19a	0.099948

Visit the online documentation for detailed code examples.

Table S3: TCGA-KIPAN Results

Model	Accuracy	F1 Weighted	F1 Macro	Recall	Precision	AUC	AUPR
SAGE	0.762 +/- 0.025	0.763 +/- 0.025	0.738 +/- 0.027	0.741 +/- 0.029	0.738 +/- 0.027	0.800 +/- 0.015	0.693 +/- 0.029
Logistic Regression	0.756 +/- 0.045	0.749 +/- 0.046	0.715 +/- 0.052	0.708 +/- 0.049	0.734 +/- 0.055	0.798 +/- 0.035	0.690 +/- 0.049
XGBoost	0.757 +/- 0.030	0.750 +/- 0.032	0.716 +/- 0.038	0.708 +/- 0.037	0.732 +/- 0.036	0.791 +/- 0.026	0.663 +/- 0.046
SVM	0.758 +/- 0.049	0.750 +/- 0.049	0.715 +/- 0.055	0.707 +/- 0.052	0.739 +/- 0.058	0.788 +/- 0.030	0.667 +/- 0.047
Random Forest	0.755 +/- 0.028	0.737 +/- 0.035	0.694 +/- 0.043	0.683 +/- 0.038	0.744 +/- 0.039	0.799 +/- 0.018	0.680 +/- 0.029
MLP	0.746 +/- 0.032	0.740 +/- 0.031	0.706 +/- 0.033	0.700 +/- 0.031	0.720 +/- 0.036	0.790 +/- 0.024	0.645 +/- 0.039
Decision Tree	0.674 +/- 0.032	0.674 +/- 0.032	0.641 +/- 0.039	0.649 +/- 0.045	0.647 +/- 0.038	0.664 +/- 0.058	0.472 +/- 0.052

Table S3.1: TCGA-KIPAN network-GNN paring comparison

Model	Graph	Accuracy	F1 Weighted	F1 Macro	Recall	Precision	AUC	AUPR
**GAT**	pearson_12	0.746 +/- 0.032	0.748 +/- 0.029	0.723 +/- 0.029	0.729 +/- 0.028	0.723 +/- 0.029	0.781 +/- 0.025	0.673 +/- 0.042
**GAT**	similarity_22	0.755 +/- 0.034	0.755 +/- 0.032	0.729 +/- 0.035	0.731 +/- 0.036	0.732 +/- 0.036	0.798 +/- 0.030	0.675 +/- 0.056
**GCN**	pearson_12	0.746 +/- 0.027	0.747 +/- 0.026	0.720 +/- 0.030	0.722 +/- 0.033	0.721 +/- 0.030	0.783 +/- 0.028	0.668 +/- 0.041
**GCN**	similarity_22	0.744 +/- 0.044	0.745 +/- 0.040	0.719 +/- 0.041	0.722 +/- 0.037	0.722 +/- 0.043	0.778 +/- 0.031	0.668 +/- 0.052
**SAGE**	pearson_12	0.762 +/- 0.025	0.763 +/- 0.025	0.738 +/- 0.027	0.741 +/- 0.029	0.738 +/- 0.027	0.800 +/- 0.015	0.693 +/- 0.029
**SAGE**	similarity_22	0.758 +/- 0.046	0.758 +/- 0.045	0.731 +/- 0.049	0.732 +/- 0.049	0.732 +/- 0.050	0.780 +/- 0.030	0.671 +/- 0.055

3.4 S4: ROSMAP (Alzheimer's Disease) Biomarker Discovery

Cohort Summary:

Stage	Methylation	mRNA	miRNA	Clinical
Raw (features x samples)	420,132 x 741	55,889 x 642	309 x 704	18 x 3584
Data Aligned (samples x features)	521 x 420,132	521 x 55,889	521 x 309	521 x 17
Variance top 100k (Methylation only)	521 x 100,000	-	-	-
Anova-F & Random Forest Intersection k=8000	521 x 772	521 x 2054	-	-
After feature selection	521 x 772	521 x 2054	521 x 309	521 x 17
Final feature selection (Variance k=300)	521 x 300	521 x 300	521 x 300	521 x 17

Target Definition: Multi-class cognitive diagnosis derived from clinical cogdx scores.

No Cognitive Impairment (NCI, n=168), Mild Cognitive Impairment (MCI, n=134), and Dementia (n=219).

Network Construction & Embedding Generation: Multi-omic networks were constructed and processed using the DPMON module with default parameters to generate latent feature embeddings, without performing downstream phenotype prediction.

Network	Function	Parameters	GNN Pairing
Cosine Similarity	similarity_network	$metric$="cosine", k=10	GAT (default)

Subnetwork Discovery & Hub Analysis:

Application of the Hybrid Louvain clustering module identified five distinct subnetworks. Graph Attention Network (GAT) embeddings were generated for the largest module to visualize the topological structure of the latent space. The smaller, highly dense subnetworks were mapped as discrete interaction graphs to identify specific regulatory hubs and high-degree drivers of disease pathology.

Figure S4.1: Embedding space 900 Multi-omic Network. GAT separation across omics types forming a biological hierarchy.

The following clusters represent subnetworks of highly correlated omics features, visualized using force-directed graphs to aid interpretation. The weight threshold is an adjustable parameter used to filter weak edges for visual clarity. Biologically, these modules reveal distinct mechanisms of Alzheimer's pathology, collectively mapping the multi-layered breakdown of the AD brain [1-8,15].

- 50 node subnetwork: Highlights synaptic plasticity and cytoskeletal scaffolding (driven by MYO10 and HOMER3).
- 25 node subnetwork: Captures white matter degeneration and cellular senescence (OLIG1, FOXO4).
- 12 node subnetwork: Identifies a critical failure in metabolic cofactor recycling and aberrant cell cycle re-entry (QDPR, CDK18).
- 6 node subnetwork: Points toward protein clearance and lysosomal dysfunction.

Table S4.1 HybridLouvain subnetwork identification

Iteration	Nodes Remaining	Correlation
0	600	0.0857
1	50	0.1563
2	25	0.1608
3	12	0.1554
4	6	0.1921

Figure S4.1 Embedding space 900 Multi-omic Network.

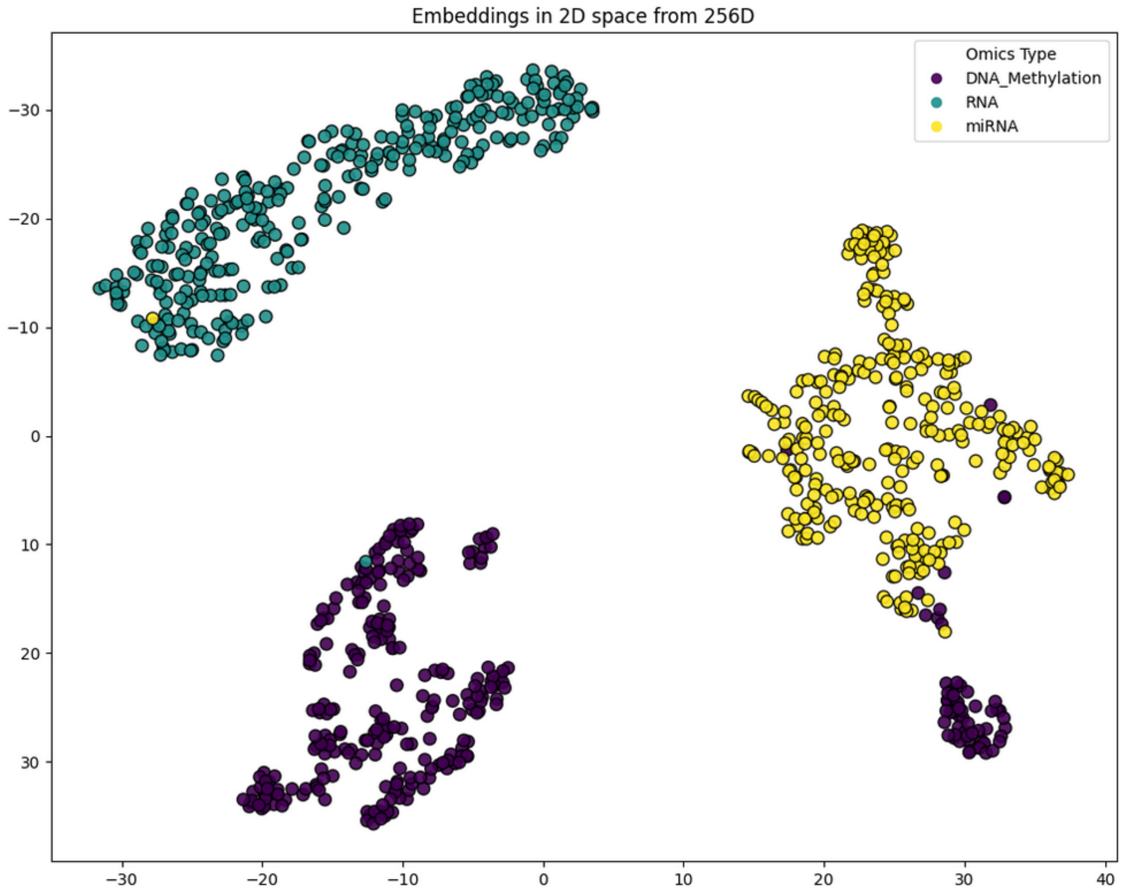

Figure S4.2 Network visualization of the 50-node "NCI vs Rest" submodule filtered at a 0.7 weight threshold.

```
nci_adj = louvain_to_adjacency(nci_submodule)
nci_mapping = plot_network(nci_adj, weight_threshold=0.7, show_labels=True, show_edge_weights=False)
print(nci_mapping)
```

```
NCI vs Rest submodule: 50 features
```

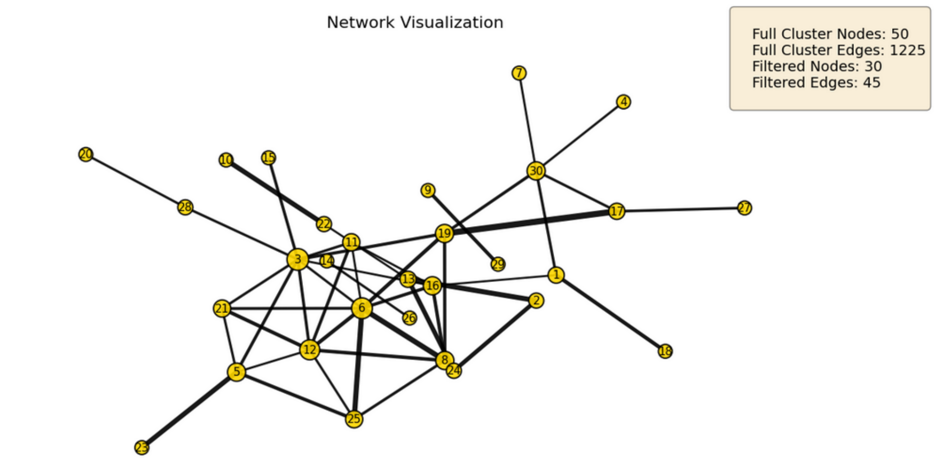

Index	Omic	Degree
3	ENSG00000145555.10	9
6	ENSG00000051128.13	8
12	ENSG00000183773.10	7
16	ENSG00000213722.4	5
8	ENSG00000180900.12	5
19	ENSG00000129250.7	5
30	ENSG00000165424.6	5
5	ENSG00000130787.9	5

Figure S4.3. Network visualization of the 25-node "NCI vs Rest" subgraph filtered at a 0.6 weight threshold.

```
nci_adj = louvain_to_adjacency(nci_submodule)
nci_mapping = plot_network(nci_adj, weight_threshold=0.6, show_labels=True, show_edge_weights=False)
print(nci_mapping)
```

```
NCI vs Rest submodule: 25 features
```

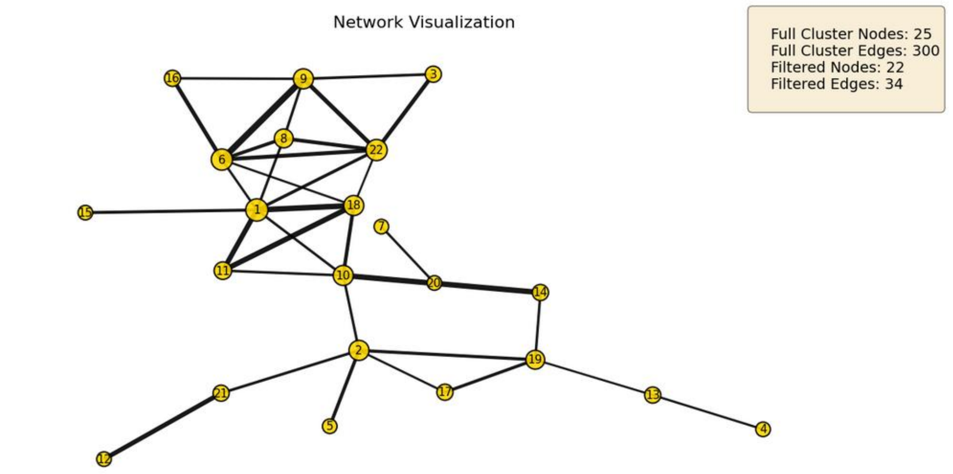

Index	Omic	Degree
1	ENSG00000198121.8	7
6	ENSG00000184481.11	6
22	ENSG00000165424.6	6
18	ENSG00000135720.8	5
9	ENSG00000129250.7	5
10	ENSG00000136541.10	5
2	ENSG00000250722.1	5

Figure S4.4. Network visualization of the 12-node "NCI vs Rest" subgraph filtered at a 0.45 weight threshold.

```
nci_mapping = plot_network(nci_adj, weight_threshold=.45, show_labels=True, show_edge_weights=False)
print(nci_mapping)
```

```
NCI vs Rest submodule: 12 features
```

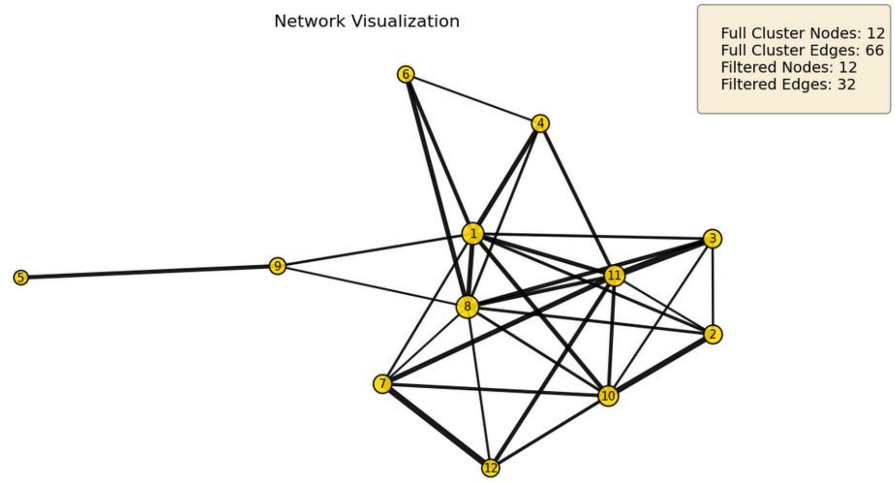

Index	Omic	Degree
8	ENSG00000115468.7	10
1	ENSG00000250722.1	9
11	ENSG00000136541.10	8
10	ENSG00000151552.7	7
2	ENSG00000117266.11	5
3	ENSG00000267548.1	5
7	ENSG00000135720.8	5

Figure S4.5 Network visualization of the 6-node "NCI vs Rest" subgraph filtered at a 0-weight threshold.

```
nci_mapping = plot_network(nci_adj, weight_threshold=0, show_labels=True, show_edge_weights=False)
print(nci_mapping)
```

```
NCI vs Rest submodule: 6 features
```

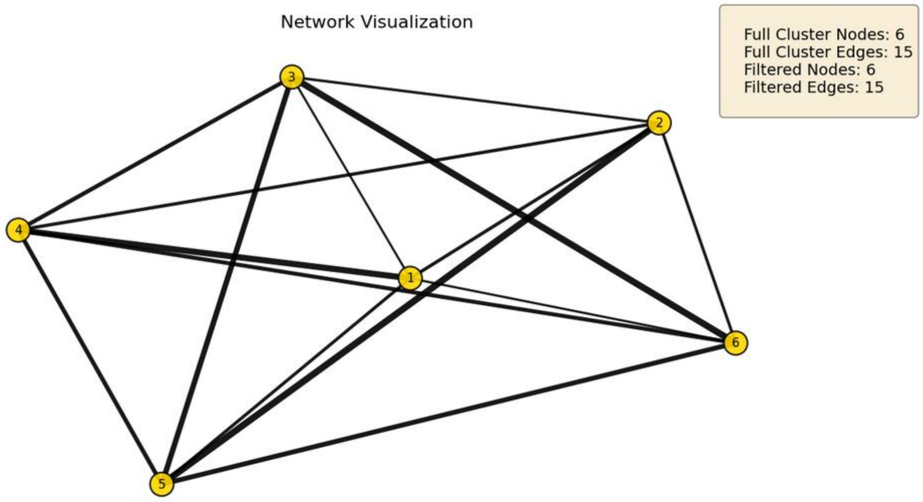

Index	Omic	Degree
1	ENSG00000117266.11	5
2	ENSG00000267548.1	5
3	ENSG00000135720.8	5
4	ENSG00000151552.7	5
5	ENSG00000136541.10	5
6	ENSG00000184007.11	5

References

1. Baar MP, Brandt RM, Putavet DA, Klein JD, Derks KW, Bourgeois BR, et al. Targeted apoptosis of senescent cells restores tissue homeostasis in response to chemotoxicity and aging. Cell. 2017;169(1):132–147.

2. Foong J, Parry SW, McNulty S. The role of tetrahydrobiopterin (BH4) metabolism in Alzheimer's disease. J Neurochem. 2020;152(S1):125–139.

3. Herrup K, Yang Y. Cell cycle regulation in the postmitotic neuron: oxymoron or new biology? Nat Rev Neurosci. 2007;8(5):368–378.

4. Herskovits AZ, Davies P. The regulation of tau phosphorylation by PCTAIRE 3: implications for the pathogenesis of Alzheimer's disease. Neurobiol Dis. 2006;23(2):398–408.

5. Mathys H, Davila-Velderrain J, Peng Z, Gao F, Mohammadi S, Young JZ, et al. Single-cell transcriptomic analysis of Alzheimer's disease. Nature. 2019;570(7761):332–337.

6. Nixon RA. The role of autophagy in neurodegenerative disease. Nat Med. 2013;19(8):983–997.

7. Parisiadou L, Bethani I, Michaki V, Krousti K, Rapti G, Efthimiopoulos S. Homer2 and Homer3 interact with amyloid precursor protein and inhibit Aβ production. Neurobiol Dis. 2008;30(3):353–364.

8. Penzes P, Vanleeuwen JE. Impaired regulation of dendritic spines in Alzheimer's disease. Alzheimers Res Ther. 2011;3(6):35.

9. Davis CF, Ricketts CJ, Wang M, Yang L, Cherniack AD, Shen H, et al. The somatic genomic landscape of chromophobe renal cell carcinoma. Cancer Cell. 2014;26(3):319–330.

10. Frew IJ, Moch H. A clearer view of the molecular complexity of clear cell renal cell carcinoma. Annu Rev Pathol. 2015;10:263–289.

11. Picard N, et al. Dmrt2 and Hmx2 direct intercalated cell diversity in the mammalian kidney through antagonistic and supporting regulatory processes. Proc Natl Acad Sci U S A. 2025;122(20). doi:10.1073/pnas.2418471122.

12. Schrader AJ, Rauer-Müller JM, Valadez-Banet S, Kuczyk MA, Hofmann R. SLC26A7 is a diagnostic marker of chromophobe renal cell carcinoma. Urol Oncol. 2016;34(10):486.e1.

13. Uhlén M, Fagerberg L, Hallström BM, Lindskog C, Oksvold P, Mardinoglu A, et al. Tissue-based map of the human proteome. Science. 2015;347(6220):1260419.

14. Vergho D, Kneitz S, Rosenwald A, Schartl M, Spahn M, Riedmiller H, et al. Combination of expression of CK7 and CLCN5 and CLCNK as a specific marker for the diagnosis of chromophobe renal cell carcinoma. Urology. 2012;79(2):484.e1.

15. AD Knowledge Portal. Available from: https://adknowledgeportal.org/. Data generated from postmortem brain tissue provided by the Religious Orders Study and Rush Memory and Aging Project (ROSMAP) cohort at Rush Alzheimer's Disease Center, Rush University Medical Center, Chicago. Supported by Cure Alzheimer's Fund and NIH grants AG058002, AG062377, NS110453, NS115064, AG062335, AG074003, NS127187, MH119509, HG008155, RF1AG062377, RF1AG054321, R01AG054012, and GM087237.
